# Dysanapsis is differentially related to lung function trajectories with distinct structural and functional patterns in COPD and variable risk for adverse outcomes

**DOI:** 10.1016/j.eclinm.2023.102408

**Published:** 2024-01-05

**Authors:** James C. Ross, Raul San José Estépar, Sam Ash, Carrie Pistenmaa, MeiLan Han, Surya P. Bhatt, Sandeep Bodduluri, David Sparrow, Jean-Paul Charbonnier, George R. Washko, Alejandro A. Diaz

**Affiliations:** a Department of Radiology, Brigham and Women's Hospital, Harvard Medical School, Boston, MA, USA; b Department of Medicine, Brigham and Women's Hospital, Harvard Medical School, Boston, MA, USA; c Division of Pulmonary and Critical Care Medicine, University of Michigan, Ann Arbor, MI, USA; d Division of Pulmonary, Allergy and Critical Care Medicine; University of Alabama at Birmingham, Birmingham, AL, USA; e VA Normative Aging Study, Veterans Affairs Boston Healthcare System, Boston, MA, USA; f Department of Medicine, Boston University Chobanian and Avedisian School of Medicine, Boston, MA, USA; g Thirona, Nijmegen, Netherlands

**Keywords:** Chronic obstructive pulmonary disease, Lung, Anatomic variation, Lung function trajectories, Dysanapsis

## Abstract

**Background:**

Abnormal lung function trajectories are associated with increased risk of chronic obstructive pulmonary disease (COPD) and premature mortality; several risk factors for following these trajectories have been identified. Airway under-sizing dysanapsis (small airway lumens relative to lung size), is associated with an increased risk for COPD. The relationship between dysanapsis and lung function trajectories at risk for adverse outcomes of COPD is largely unexplored. We test the hypothesis that dysanapsis differentially affects distinct lung function trajectories associated with adverse outcomes of COPD.

**Methods:**

To identify lung function trajectories, we applied Bayesian trajectory analysis to longitudinal FEV1 and FVC Z-scores in the COPDGene Study, an ongoing longitudinal study that collected baseline data from 2007 to 2012. To ensure clinical relevance, we selected trajectories based on risk stratification for all-cause mortality and prospective exacerbations of COPD (ECOPD). Dysanapsis was measured in baseline COPDGene CT scans as the airway lumen-to-lung volume (a/l) ratio. We compared a/l ratios between trajectories and evaluated their association with trajectory assignment, controlling for previously identified risk factors. We also assigned COPDGene participants for whom only baseline data is available to their most likely trajectory and repeated our analysis to further evaluate the relationship between trajectory assignment and a/l ratio measures.

**Findings:**

We identified seven trajectories: supranormal, reference, and five trajectories at increased risk for mortality and exacerbations. Three at-risk trajectories are characterized by varying degrees of concomitant FEV1 and FVC impairments and exhibit airway predominant COPD patterns as assessed by quantitative CT imaging. These trajectories have lower a/l ratio values and increased risk for mortality and ECOPD compared to the reference trajectory. Two at-risk trajectories are characterized by disparate levels of FEV1 and FVC impairment and exhibit mixed airway and emphysema COPD patterns on quantitative CT imaging. These trajectories have markedly lower a/l ratio values compared to both the reference trajectory and airway-predominant trajectories and are at greater risk for mortality and ECOPD compared to the airway-predominant trajectories. These findings were observed among the participants with baseline-only data as well.

**Interpretation:**

The degree of dysanapsis appears to portend patterns of progression leading to COPD. Assignment of individuals—including those without spirometric obstruction—to distinct trajectories is possible in a clinical setting and may influence management strategies. Strategies that combine CT-assessed dysanapsis together with spirometric measures of lung function and smoke exposure assessment are likely to further improve trajectory assignment accuracy, thereby improving early detection of those most at risk for adverse outcomes.

**Funding:**

United States National Institute of Health, 10.13039/100008184COPD Foundation, and 10.13039/100005292Brigham and Women's Hospital.

**Research in context**

**Evidence before this study**

Previous work has established that there are various lung function trajectories throughout the life course; several of these lead to COPD, including accelerated decline and failure to achieve peak lung function in early adulthood. Other work has identified CT-assessed dysanapsis—a mismatch of airway lumen caliber and lung volume—as a risk factor for COPD. However, the relationship between dysanapsis and lung function trajectories is largely unexplored.

**Added value of this study**

We applied a Bayesian trajectory approach to longitudinal data from the COPDGene study to *jointly* model the coevolution of FEV1 and FVC in relation to age and cigarette smoke exposure. Application of this approach to the COPDGene data identified seven lung function trajectories, five of which are at increased risk for exacerbations and mortality. We show that moderate levels of dysanapsis are related to airway predominant trajectories, while more pronounced dysanapsis is related to mixed airway and emphysema trajectories.

**Implications of all the available evidence**

We show that airway under sizing dysanapsis (small airway lumens relative to lung size) is not only a risk factor for COPD, but that the degree of dysanapsis—given its association with trajectory assignment—appears to portend patterns of progression with distinct structural and functional patterns with variable risk for adverse outcomes. Assigning individuals—including those without obstruction—to distinct trajectories could enable earlier, more tailored preventive and management strategies. Strategies that combine CT-assessed dysanapsis together with spirometric measures of lung function and smoke exposure assessment are likely to further improve trajectory assignment accuracy, thereby improving early detection of those most at risk for adverse outcomes.

## Introduction

Recent work has identified distinct lung function trajectories throughout the life-course, and abnormal trajectories are associated with increased risk of developing chronic obstructive pulmonary disease (COPD) and with premature mortality.^1^^,^^2^ There is significant interest in identifying and understanding the risk factors associated with abnormal lung function trajectories. Factors linked to lung function life-course trajectories include genotypes, tobacco exposure, and allergic sensitization.^3^, ^4^, ^5^ However, whether anatomical characteristics of the lung associate with lung function trajectories is less known.

Dysanapsis is one such anatomical feature of the lung and is defined as a mismatch between airway lumen caliber and lung volume. Smith and colleagues showed that dysanapsis as measured on computed tomography (CT) was significantly associated with COPD among older adults, with lower airway tree caliber relative to lung size associated with greater risk.^6^ Forno and colleagues demonstrated that dysanapsis is associated with exacerbations in obese children with asthma.^7^ Furthermore, Bhatt and colleagues reported that airway lumens (adjusted for lung size) were smaller in women than in men, and this conferred a greater risk for morbidity and mortality.^8^ Here we sought to bridge the knowledge gap between dysanapsis as an anatomical feature of the lung and its relationship with lung function trajectories linked to distinct structural and functional patterns of COPD.

## Methods

### Study cohort

The COPDGene Study is an ongoing, multicenter, longitudinal study designed to investigate the genetic and epidemiologic characteristics of COPD.^9^ COPDGene enrolled 10,198 non-Hispanic white and African American ever-smokers with and without COPD and 454 never-smokers. Participants were between the ages of 45 and 80 years and ever-smokers had a minimum of 10 pack-years cigarette smoke exposure at baseline (a small number of participants less than 45 years old at baseline were also recruited and considered in our study). Demographic information including age, sex, race, height, and body mass index (BMI) was obtained with standardized questionnaires and procedures. Information about parental characteristics—smoking history and history of emphysema, COPD, chronic bronchitis, and asthma—was recorded at baseline. Baseline data collection procedures were repeated at approximately 5 years (visit 2) and 10 years (visit 3); acquisition of 10-year follow-up data is ongoing. Study data consisting of smoking history (pack-years exposure and current smoking status), post-bronchodilator spirometric measures of lung function, volumetric CT of the chest, history of gastroesophageal reflux disease (GERD), health-related quality of life as measured by the St. George's Respiratory Questionnaire (SGRQ), exercise capacity as measured by the 6-min walk test (6MWD), and dyspnea assessed with the modified Medical Research Council (mMRC) scale are recorded at each study visit. Spirometry and the 6-min walk test were performed per ATS recommendations.^10^ The BODE index (BMI, airflow Obstruction, Dyspnea and Exercise capacity) was computed for each visit. Post-bronchodilator forced expiratory volume in 1 s (FEV_1_), forced vital capacity (FVC), and their percent predicted values were used to define spirometric groups (COPD GOLD groups 1–4). The institutional review boards of all participating centers approved the COPDGene Study, and all participants provided written informed consent.

### Trajectory analysis

Our trajectory analysis approach used a combination of prior knowledge, data-driven inference, and model selection based on clinical relevance (using analysis of mortality and exacerbation risk). We used the *bayes_traj* (https://github.com/acil-bwh/bayes_traj) software routine, which is a Bayesian version of Group Based Trajectory Modeling (GBTM) and enables incorporation of prior information into the data fitting process.^11^ We used GLI reference equations—which account for age, height, sex, and race—to compute FEV1 and FVC Z-scores.^12^ Investigators have commonly performed lung function trajectory analysis using functions of age as predictors. We additionally included smoke exposure and smoking status terms given that COPDGene is comprised of current and former smokers, and because it is generally accepted that smokers have differential response to smoke exposure. By including these terms in the set of predictors, individuals assigned to a given trajectory are expected to have similar patterns of progression over time and in response to smoke exposure and smoking status. Before performing trajectory analysis, we centered the age variable at 20 for men and 18 for women; this makes the intercept term interpretable as the Z-score value obtained at the approximate age of peak lung function. We refer to the centered age variable as “years since presumed peak lung function”. We evaluated several candidate predictor sets as described in the supplement. Selected trajectory model predictors included an intercept term, years since presumed peak lung function (years since presumed peak lung function),^1^ pack-years smoke exposure, and an interaction between smoking status and years since presumed peak lung function.

We restricted trajectory modeling to those participants for whom two or more longitudinal time points are available, and we excluded data corresponding to pack-year smoke exposure exceeding 150 pack-years (less than 1% of the data sample). We used the *bayes_traj* routine to jointly model FEV1 and FVC Z-score trajectories. The rationale for simultaneously considering both FEV1 and FVC Z-scores was to more fully represent heterogeneity in spirometric progression patterns. Additionally, by jointly considering these two measures, the ability to detect smaller groups with distinct spirometric progression patterns is improved owing to the greater number of measures per participant being modeled. The Bayesian paradigm enables incorporation of prior belief in the form of probabilistic *priors*. Since Z-score values are, by definition, normally distributed around a mean of zero, we used zero-centered normal distributions for the FEV1 and FVC Z-score intercept coefficients. This was intended to mitigate the effects of data absence in early adulthood (due to COPDGene enrollment criteria requiring participants to be at least 45 years old).

We executed the trajectory routine with several hundred random initializations, and we sorted the resulting models according to the Watanabe-Akaike information criterion (WAIC2).^13^ We focused on those trajectories that account for at least 2% of the data sample. To select clinically meaningful trajectories for further analysis, we evaluated models with the best WAIC2 scores in terms of exacerbation and mortality risk stratification, and we selected the model with statistically significant discrimination between adjacent trajectories in terms of mortality hazard ratios and exacerbation incident ratio ratios (See supplement for additional details).

For each participant, trajectory modeling produced a probability of assignment to each of the identified trajectories. We assigned each participant to their most probable trajectory and treated this assignment as a factor variable for further analysis. We then used the derived trajectory model to assign those COPDGene participants for whom only baseline data is available (the “baseline-only” cohort) to their most probable trajectory. These participants were not included in the data sample used to train the trajectory model and enabled us to further evaluate the relationship between trajectory assignment and a/l ratio values. Figure E1 describes the data selection procedure.

### Outcomes

We analyzed two outcomes to assess the clinical meaningfulness of trajectories: exacerbations during follow-up and all-cause mortality. Exacerbations are defined in COPDGene as a new onset of or increase in cough, phlegm, or dyspnea. An episode that requires antibiotics and/or steroids is counted as an exacerbation. Participants were asked every three to six months about exacerbation episodes through the COPDGene longitudinal follow-up program.^14^ Deaths were also identified through the longitudinal follow-up program and were confirmed with death certificates from the Social Security Death Index.

### CT analysis and dysanapsis

Chest CT analysis in the COPDGene Study has been described previously.^9^ Briefly, quantitative analysis of inspiratory CT scans using Thirona software (Thirona LungQ, Nijmegen, The Netherlands) produced measures of emphysema and airway wall thickness. The Hounsfield unit (HU) value representing the 15th percentile of the lung region HU histogram (Perc15) was used for densitometric assessment of the lung parenchyma.^15^ Airway wall thickening was assessed as the square root of the wall area of a theoretical airway with an internal lumen perimeter of 10 mm (Pi10).^16^ Additionally, we quantified dysanapsis on baseline inspiratory CT scans using airway lumen diameters at 13 anatomic locations together with the CT-assessed total lung volume (VIDA Diagnostics, Coralville, IA, USA). Airway locations included: mainstem left and right bronchi; bronchus intermedius; lobar bronchi of the left upper lobe (LUL), left lower lobe (LLL), right upper lobe (RUL), right middle lobe (RML), and right lower lobe (RLL); and the following six segmental bronchi: apicoposterior segment of LUL, medial segment of lingula, basal posterior segment of LLL, apical segment of RUL, medial segment of RML, and posterior basal segment of RLL. The geometric mean of the 13 airway lumen diameters was divided by the cube root of CT-measured total lung volume to provide a measure of dysanapsis, referred to as the airway to lung (a/l) ratio.^6^ Lower a/l ratio values indicate smaller airway tree lumens relative to lung size and thus greater dysanapsis.

### Statistical analysis

Mortality time-to-event and exacerbation count modeling was performed using the R software package (version 3.6.1).^17^ Participant trajectory assignment was treated as a factor variable. We performed extended Cox modeling (R's coxph routine^18^^,^^19^) using age as our time scale and all-cause mortality as our outcome of interest. We examined scaled Schoenfeld residuals with a two-sided chi-square test to assess the proportional hazards assumption for trajectory assignment. Our reduced model included current smoking status, pack-years smoke exposure, sex, and race as covariates; the full model also included BMI, MMRC, and 6MWD, which have shown to be independent predictors of mortality.^20^ For prospective number of total exacerbations, we used zero-inflated negative binomial mixed modeling (R's glmm.zinb routine^21^) with an offset variable to account for differences in observation times. The reduced model adjusted for age, current smoking status, pack-years smoke exposure, sex, and race; the full model additionally adjusted for SGRQ, gastroesophageal reflux, and number of exacerbations over the previous year.^22^

We assessed differences in a/l ratios between trajectories using the Mann–Whitney test (using python's scipy.stats.mannwhitneyu routine, version 1.7.3)^23^ and used Bonferroni correction for multiple comparisons.

Parental characteristics including parental emphysema, COPD, chronic bronchitis, and asthma, as well as whether parents were cigarette smokers, have been previously shown to associate with lung function trajectories.^24^ To evaluate whether the a/l ratio is an independent predictor of trajectory assignment, we considered the a/l ratio together with these parental characteristics in a multinomial logistic regression using a forward stepwise-selection strategy. The a/l ratios were scaled by the standard deviation of the reference trajectory for ease of interpretation, so that relative risk ratios can be interpreted as how many times more or less likely a trajectory assignment is to be (relative to the reference trajectory) for a unit change in standard deviation.

Finally, we investigated whether dysanapsis could be considered a static anatomical feature of the lung or one that changes with age, and we also assessed whether airway wall thickening and emphysematous destruction of the parenchyma could confound the measured a/l ratio values. To do this, we estimated the per-trajectory relationships between a/l ratio, airway wall thickening (Pi10), and emphysema (Perc15) with age using ordinary least squares regression (scipy.stats.OLS routine, version 1.7.3)^23^ (see supplement for details).

### Role of the funding source

The funders had no role in study design, data collection, data analysis, data interpretation, or writing of the report.

## Results

Table 1 and Table 2 provide characteristics of the 5401 COPDGene participants on whom trajectory modeling was performed by visit and a/l tertiles, respectively. For this sample, 538 mortality events are recorded with a median follow-up time of 10.4 years (interquartile range: 9.5–11.1 years). A total of 13,795 exacerbation events are recorded (2.63 per-participant on average, interquartile range: 0–3) with a mean follow-up time of 6.5 years per participant.

**Table 1:** Characteristics of COPDGene participants included in the study by visit.

Characteristic	Baseline	5-yr follow-up	10-yr follow-up
*N*	5401	5285	1804
Female	2699 (49)	2647 (50)	919 (50)
African American	1693 (31)	1628 (30)	553 (30)
Age, yr	60 ± 9	65 ± 9	69 ± 9
FEV1 Z-Score	−1.1 ± 1.3	−1.2 ± 1.3	−1.2 ± 1.3
FEV1% predicted	82 ± 21	80 ± 23	80 ± 24
FVC Z-Score	−0.6 ± 1.1	−0.7 ± 1.1	−0.6 ± 1.1
FVC% predicted	89 ± 16	87 ± 17	88 ± 19
FEV1/FVC Z-Score	−1.0 ± 1.5	−1.0 ± 1.5	−1.1 ± 1.5
6MWD (feet)	1431 ± 369	1306 ± 429	1292 ± 402
SGRQ	22 ± 21	22 ± 21	22 ± 20
MMRC	1.1 ± 1.3	1.1 ± 1.4	1.2 ± 1.3
MMRC score ≥2	1791 (33)	1873 (35)	669 (37)
BODE	1.2 ± 1.7	1.5 ± 2.0	1.6 ± 2.0
BODE score ≥7	74 (1)	164 (3)	58 (3)
GERD	916 (26)	595 (27)	268 (40)
BMI, kg/m^2^	29 ± 6	29 ± 6	29 ± 6
Pack-years	41 ± 23	43 ± 23	44 ± 23
Current smoker	2629 (49)	2060 (39)	624 (35)

Data are presented as mean ± SD or number (percent).

**Table 2:** Baseline characteristics of COPDGene participants by a/l ratio tertile.^a^

Characteristic	1st Tertile (more dysanapsis)	2nd Tertile	3rd Tertile (less dysanapsis)
*N*	1735	1735	1736
Female	911 (53)	865 (50)	783 (45)
African American	355 (20)	464 (27)	762 (44)
Age, yr	60 ± 9	60 ± 9	59 ± 9
FEV1 Z-Score	−1.9 ± 1.5	−1.0 ± 1.3	−0.6 ± 1.1
FEV1% predicted	69 ± 24	84 ± 21	90 ± 18
FVC Z-Score	−0.8 ± 1.2	−0.5 ± 1.1	−0.5 ± 1.1
FVC % predicted	86 ± 18	91 ± 16	91 ± 16
FEV1/FVC Z-Score	−2.1 ± 1.6	−1.0 ± 1.4	−0.3 ± 1.2
6MWD (feet)	1384 ± 364	1469 ± 358	1447 ± 373
GERD	498 (29)	440 (25)	420 (24)
MMRC	1.4 ± 1.4	1.0 ± 1.3	1.0 ± 1.3
MMRC score ≥ 2	771 (44)	510 (29)	503 (28)
SGRQ	29 ± 23	21 ± 20	19 ± 19
BODE	2.0 ± 2.1	1.1 ± 1.6	0.9 ± 1.4
BODE score ≥ 7	60 (3)	22 (1)	8 (0)
BMI, kg/m^2^	29 ± 6	29 ± 6	30 ± 6
Pack-years	46 ± 24	42 ± 22	39 ± 22
Current smoker	902 (52)	766 (44)	880 (51)
Father, emphysema	286/1286 (22)	213/1342 (16)	175/1302 (13)
Mother, emphysema	251/1475 (17)	145/1489 (10)	114/1460 (8)
Father, COPD	159/1218 (13)	130/1292 (10)	92/1252 (7)
Mother, COPD	189/1430 (13)	132/1454 (9)	91/1431 (6)
Father, chronic bronchitis	108/1215 (9)	93/1277 (7)	71/1257 (6)
Mother, chronic bronchitis	160/1442 (11)	129/1455 (9)	113/1443 (8)
Father, asthma	96/1281 (8)	71/1346 (5)	55/1301 (4)
Mother, asthma	126/1461 (9)	93/1466 (6)	129/1463 (9)
Father, smoked cigarettes	1298/1599 (81)	1265/1596 (79)	1200/1575 (76)
Mother, smoked cigarettes	986/1680 (59)	877/1677 (52)	860/1665 (52)
Mother smoked during pregnancy	455/1289 (35)	369/1316 (28)	342/1270 (27)

a a/l ratio measurements available on 5206 of the 5401 participants included in our study. Data are presented as mean ± SD, number (percent), or number/total (percent).

**Fig. 1:**
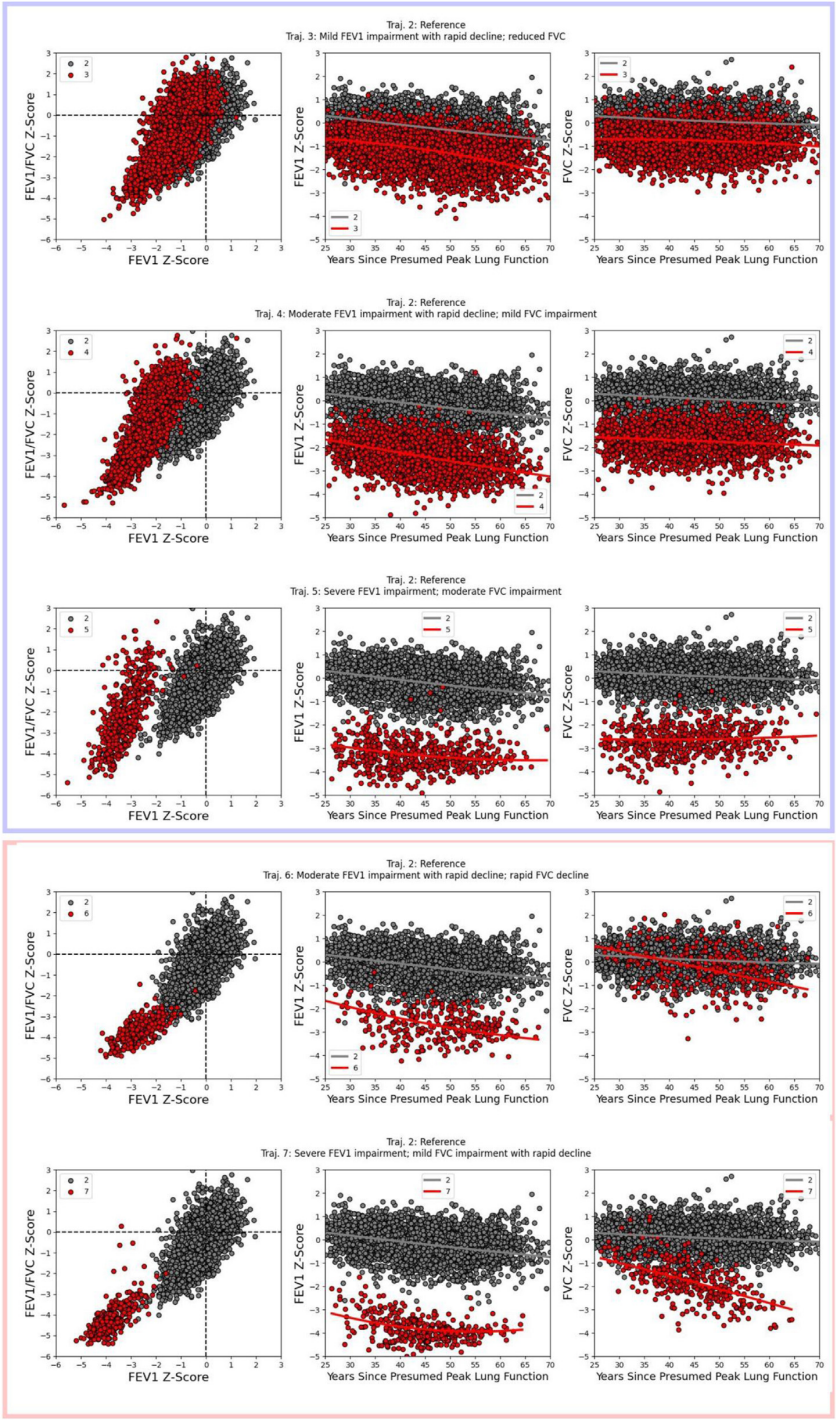
Spirometry trajectory plots in COPDGene. For each row, we show a comparison between trajectory 2 (reference trajectory in gray) and an at-risk trajectory (in red). Solid lines in the middle and right panels represent predicted values for each trajectory. The scatter plots include all available longitudinal observations per participant. The left-most panel in each row shows FEV1/FVC Z-scores vs. FEV1 Z-scores and indicates the empirically observed levels of obstruction and FEV1 impairment. Trajectories 3–5 (light blue border) are characterized by airway predominant abnormality leading to COPD; trajectories 6 and 7 (light red border) are characterized by mixed airway and emphysema abnormality. Note: Trajectory 1 (supra-normal) is not shown.

Bayesian trajectory analysis identified seven trajectories in COPDGene, with each trajectory accounting for at least 2% of the data sample. Per-trajectory participant characteristics at baseline are provided in Table 3, and trajectories are plotted in Fig. 1 (Figure E3 shows all trajectories in a single plot). Per-trajectory participant characteristics of the baseline-only cohort are provided in Table E6. Trajectory 1 is characteristic of supranormal individuals. Trajectory 2 exhibits FEV1 and FVC Z-score values within the normal range with modest declines in Z-score values with increasing age and smoke exposure; we treated this trajectory as the reference for subsequent analysis. The remaining trajectories fall into two broad categories based on qualitative assessment: those having varying degrees of concomitant FEV1 and FVC impairments (trajectories 3–5) and those having disparate levels of FEV1 and FVC impairment (trajectories 6 and 7). Referring to Table 3 shows that those in the first category exhibit greater airway wall thickening (higher Pi10 values) with no marked difference in emphysema (measured by Perc15) compared to the reference trajectory. On the other hand, trajectories 6 and 7 exhibit both greater airway wall thickening and emphysema levels. We therefore refer to the first category as airway predominant trajectories and the second category as mixed airway and emphysema trajectories. Trajectories 3–7 are further summarized as follows:

**Table 3:** Baseline characteristics of COPDGene participants by lung function trajectory.

Characteristic	1	2	3	4	5	6	7
*N*	420 (7.8)	1519 (28.1)	**1957 (36.2)**	**958 (17.7)**	**265 (4.9)**	*134 (2.5)*	*148 (2.7)*
Female	201 (47)	759 (49)	**997 (50)**	**471 (49)**	**128 (48)**	*66 (49)*	*77 (52)*
African American	121 (28)	431 (28)	**633 (32)**	**365 (38)**	**92 (34)**	*26 (19)*	*25 (16)*
Age, yr	60 ± 9	60 ± 9	**60 ± 9**	**59 ± 9**	**59 ± 8**	*63 ± 8*	*60 ± 7*
FEV1 Z-Score	0.8 ± 0.6	−0.1 ± 0.6	**−1.1 ± 0.7**	**−2.3 ± 0.7**	**−3.4 ± 0.6**	*−2.5 ± 0.7*	*−3.6 ± 0.6*
FEV1% predicted	111 ± 9	98 ± 10	**82 ± 11**	**63 ± 14**	**46 ± 12**	*59 ± 13*	*42 ± 13*
FVC Z-score	1.0 ± 0.5	0.1 ± 0.6	**−0.7 ± 0.6**	**−1.6 ± 0.6**	**−2.7 ± 0.6**	*0.0 ± 0.8*	*−1.4 ± 0.8*
FVC% predicted	112 ± 9	100 ± 9	**87 ± 9**	**74 ± 9**	**60 ± 8**	*98 ± 12*	*77 ± 13*
FEV1/FVC Z-Score	−0.3 ± 0.8	−0.4 ± 1.0	**−0.7 ± 1.2**	**−1.5 ± 1.5**	**−2.2 ± 1.5**	*−3.6 ± 0.6*	*−4.1 ± 0.8*
BMI, kg/m^2^	28 ± 5	28 ± 5	**30 ± 6**	**31 ± 7**	**32 ± 7**	*27 ± 5*	*27 ± 6*
Pack-years	42 ± 25	41 ± 24	**40 ± 22**	**42 ± 23**	**44 ± 24**	*47 ± 19*	*43 ± 17*
Pi10	1.8 ± 0.4	2.0 ± 0.4	**2.2 ± 0.5**	**2.6 ± 0.6**	**2.9 ± 0.6**	*2.6 ± 0.5*	*2.7 ± 0.5*
Perc15	−917 ± 21	−912 ± 23	**−908 ± 26**	**−909 ± 34**	**−913 ± 40**	*−945 ± 21*	*−950 ± 23*
6MWD (feet)	1588 ± 331	1533 ± 344	**1451 ± 349**	**1277 ± 358**	**1134 ± 384**	*1370 ± 317*	*1229 ± 339*
BODE	0.4 ± 0.8	0.5 ± 1.0	**0.9 ± 1.3**	**2.2 ± 1.9**	**4.0 ± 2.1**	*2.3 ± 1.7*	*4.3 ± 1.8*
MMRC	0.5 ± 0.9	0.7 ± 1.1	**1.0 ± 1.3**	**1.6 ± 1.4**	**2.3 ± 1.3**	*1.7 ± 1.3*	*2.4 ± 1.2*
CT Lung Vol.	7.54 ± 1.1	6.9 ± 1.0	**6.33 ± 0.9**	**6.16 ± 1.1**	**5.86 ± 1.1**	*7.8 ± 1.0*	*7.75 ± 1.0*
COPD	59 (14)	297 (19)	**620 (31)**	**522 (54)**	**194 (73)**	*134 (100)*	*147 (99)*

Data are presented as mean ± SD or number (percent). Trajectories 1 and 2 are supranormal and reference, respectively. Trajectories 3–5 (bold) are characterized by airway predominant abnormality leading to COPD; trajectories 6 and 7 (italic) are characterized by mixed airway and parenchymal abnormality.

Airway predominant trajectories:

• Trajectory 3: mild FEV1 impairment with rapid decline; reduced FVC
• Trajectory 4: moderate FEV1 impairment with rapid decline; mild FVC impairment
• Trajectory 5: severe FEV1 impairment; moderate FVC impairment

Mixed airway and emphysema trajectories:

• Trajectory 6: moderate FEV1 impairment with rapid decline; rapid FVC decline
• Trajectory 7: severe FEV1 impairment; mild FVC impairment with rapid decline

3762 COPDGene participants comprised the baseline-only cohort. Of these, 3194 (85%) were assigned to one of the seven trajectories described above. For this subset, 1063 mortality events are recorded with a median follow-up time of 3.9 years (interquartile range: 2.1–6.1 years). A total of 3675 exacerbation events are recorded (1.2 per-participant on average) with a mean follow-up time of 3 years per participant.

Table 4 shows significantly increased all-cause mortality hazard ratios for each of the at-risk trajectories compared to trajectory 2, as well as significantly increased incident rate ratios for exacerbations for each of the at-risk trajectories using trajectory group 2 as the reference group. These results were significant in the reduced model and in the full model (Tables E2–E5 provide hazard ratios and incident rate ratios with the at-risk trajectories taken as the reference). Table 4 also shows hazard ratios and incident rate ratios corresponding to those participants in the baseline-only cohort that were assigned to their most likely trajectories.

**Table 4:** Hazard ratios for all-cause mortality and incident rate ratios for the total number of exacerbations during follow-up by trajectory.

Trajectory modeling cohort								
Trajectory	Reduced models				Full models			
	HR	p-value	IRR	p-value	HR	p-value	IRR	p-value
1	0.72 (0.45, 1.16)	0.17	0.86 (0.69, 1.08)	0.20	0.83 (0.52, 1.35)	0.46	0.90 (0.72, 1.11)	0.32
**3**	**1.20 (0.93, 1.55)**	**0.16**	**1.63 (1.42, 1.86)**	**<0.001**	**1.06 (0.82, 1.38)**	**0.65**	**1.49 (1.31, 1.69)**	**<0.001**
**4**	**2.50 (1.95, 3.21)**	**<0.001**	**3.34 (2.86, 3.89)**	**<0.001**	**1.48 (1.13, 1.94)**	**0.005**	**2.61 (2.25, 3.02)**	**<0.001**
**5**	**4.33 (3.10, 6.04)**	**<0.001**	**6.40 (5.07, 8.06)**	**<0.001**	**1.98 (1.36, 2.87)**	**<0.001**	**4.25 (3.41, 5.30)**	**<0.001**
*6*	*2.96 (1.94, 4.52)*	*<0.001*	*4.90 (3.60, 6.66)*	*<0.001*	*1.73 (1.12, 2.69)*	*0.01*	*3.59 (2.69, 4.79)*	*<0.001*
*7*	*7.43 (5.28, 10.44)*	*<0.001*	*10.96 (8.25, 14.57)*	*<0.001*	*2.18 (1.47, 3.24)*	*<0.001*	*6.63 (5.07, 8.67)*	*<0.001*
Baseline-only cohort								

Trajectory	Reduced models				Full models			
	HR	p-value	IRR	**p-value**	**HR**	**p-value**	**IRR**	**p-value**

1	1.09 (0.76, 1.56)	0.64	0.98 (0.71, 1.34)	0.89	1.12 (0.78, 1.60)	0.55	1.07 (0.78, 1.46)	0.69
**3**	**1.42 (1.17, 1.73)**	**<0.001**	**1.61 (1.32, 1.96)**	**<0.001**	**1.32 (1.08, 1.62)**	**0.006**	**1.30 (1.07, 1.57)**	**0.009**
**4**	**1.88 (1.54, 2.29)**	**<0.001**	**3.26 (2.63, 4.04)**	**<0.001**	**1.52 (1.23, 1.88)**	**<0.001**	**1.79 (1.44, 2.23)**	**<0.001**
**5**	**2.37 (1.87, 3.00)**	**<0.001**	**4.84 (3.52, 6.66)**	**<0.001**	**1.74 (1.33, 2.26)**	**<0.001**	**2.02 (1.46, 2.80)**	**<0.001**
*6*	*1.99 (1.52, 2.62)*	*<0.001*	*6.07 (4.13, 8.92)*	*<0.001*	*1.53 (1.14, 2.06)*	*0.005*	*3.24 (2.21, 4.76)*	*<0.001*
*7*	*3.04 (2.39, 3.86)*	*<0.001*	*13.36 (9.78, 18.26)*	*<0.001*	*2.02 (1.54, 2.64)*	*<0.001*	*4.96 (3.58, 6.88)*	*<0.001*

Reduced models (left): extended Cox models adjusted for pack-years smoke exposure, current smoking status, sex, and race; zero-inflated negative binomial mixed models adjusted for age, pack-years smoke exposure, current smoking status, sex, and race. Full models (right): extended Cox models adjusted for pack-years smoke exposure, current smoking status, BMI, MMRC, 6MWD, sex, and race; zero-inflated negative binomial mixed models adjusted for age, sex, race, pack-years smoke exposure, current smoking status, SGRQ, GERD, and number of exacerbations in the previous year. Trajectory 2 is used as the reference in all models. Trajectories 3–5 (bold) are characterized by airway predominant abnormality leading to COPD; trajectories 6 and 7 (italic) are characterized by mixed airway and parenchymal abnormality.

**Fig. 2:**
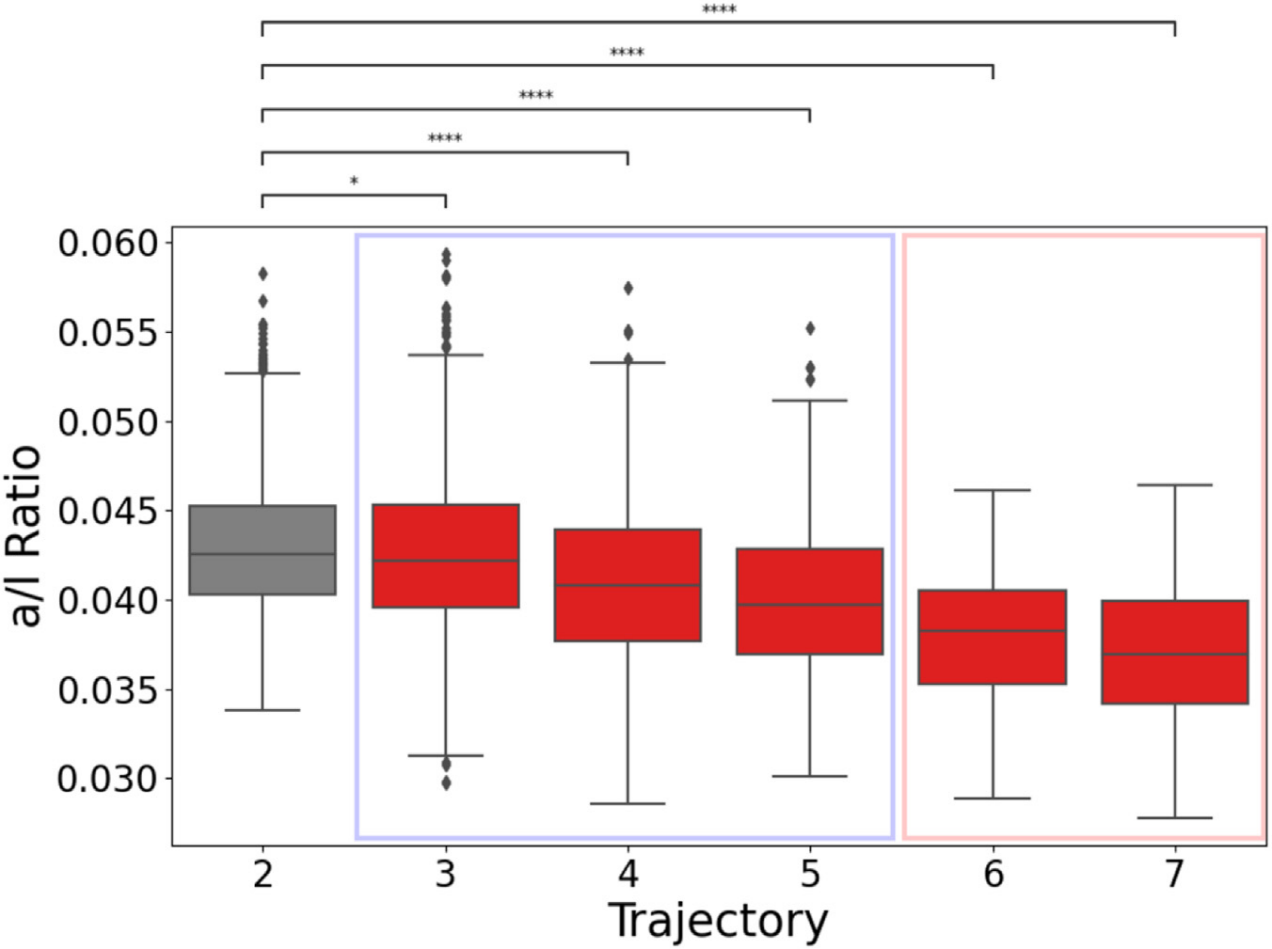
Boxplot showing a/l ratios of reference trajectory 2 (gray) and trajectories at increased risk of all-cause mortality and exacerbations (trajectories 3–7 in red). Trajectories 3–5 (light blue border) are characterized by airway predominant abnormality leading to COPD; trajectories 6 and 7 (light red border) are characterized by mixed airway and emphysema abnormality. Indicated above are p-values corresponding to pairwise statistical comparisons between trajectory 2 and each at-risk trajectory (using Mann–Whitney test and Bonferroni correction for multiple comparisons): ∗p < 0.05, ∗∗∗∗p < 0.0001.

In Fig. 2 we compare a/l ratio measures between the reference trajectory and at-risk trajectories. Within each trajectory category (airway predominant and mixed airway and emphysema) there is a trend toward lower a/l ratio values with increasing risk for all-cause mortality and prospective exacerbations. Notably, while all at-risk trajectories exhibit lower a/l ratio values compared to the reference trajectory, decrements are less pronounced in the airway predominant category compared to the mixed airway and emphysema category. These results are also evident in the baseline-only cohort (Figure E5). We also considered the statistical significance of a/l ratio values between adjacent at-risk trajectories. In the training cohort, we observed a significant difference between trajectories 3 and 4 (p < 1.00e-04) and between 5 and 6 (p < 1.00e-03), but not between 4 and 5 or 6 and 7. In the baseline-only cohort, we observed a significant difference between trajectories 3 and 4 (p < 1.00e-04), between 4 and 5 (p < 0.01), and between 5 and 6 (p < 1.00e-04), but not between 6 and 7.

Table 5 shows that dysanapsis is associated with increased relative risk ratios (RRR) for assignment to each of the at-risk trajectories, with the magnitude of the RRR increasing from trajectory 3 to 7. Using forward stepwise selection, we find that risk ratios corresponding to scaled a/l ratios remain significant after adjusting for parental risk factors.

**Table 5:** Multinomial logistic regression analysis for the association between dysanapsis and trajectory assignment.

Trajectories	RRR	[95% Conf. interval]		p-value
1 vs. 2	0.91	0.82	1.01	0.07
3 vs. 2	1.09	1.03	1.17	0.006
4 vs. 2	1.52	1.40	1.65	<0.001
5 vs. 2	1.84	1.60	2.12	<0.001
6 vs. 2	3.22	2.66	3.90	<0.001
7 vs. 2	3.99	3.28	4.84	<0.001

Analysis dependent variable is trajectory assignment; independent variables include an intercept term and a/l ratio values scaled by 0.0039 (the a/l standard deviation of the reference trajectory 2). RRRs correspond to a 1-standard deviation decrease in a/l ratio.

## Discussion

We analyzed longitudinal data from the COPDGene Study and identified seven distinct lung-function trajectories. A benefit of trajectory analysis is that it identifies population subgroups with similar progression patterns in response to age and external factors, such as smoke exposure. Similarity in progression patterns putatively indicates endotypes, population subgroups that respond and progress in a similar fashion due to underlying similarities in genetics, physiology, or—as suggested here—anatomic architecture. Indeed, previous research demonstrates a genetic basis for airway branch variation.^25^ Our analysis found that an anatomical characteristic of the lung, dysanapsis, is associated with the more at-risk lung-function trajectories, which are at increased risk for all-cause mortality and prospective pulmonary exacerbations. Interestingly, other anatomical features—airway fractal dimension and total airway count—have been associated with lung function independent of dysanapsis in healthy participants.^26^ We posit that these, too, may be linked to various lung function trajectories.

Nearly all participants assigned to trajectories 6 and 7 had COPD at baseline and—as noted above—also had more emphysema (lower Perc15 values) and higher Pi10 values compared to trajectory 2. Emphysema is typically thought to accompany a rapid decline in FEV1; this is seen in trajectory 6 but not in trajectory 7. Indeed, in trajectory 7 decline in FEV1 appears to halt in older age. Whether this trend reflects response to intervention, survival bias, or some other factor requires further investigation. The rapid decline in FVC in trajectories 6 and 7 might reflect an increasing degree of air trapping, which is supported by the high CT lung volumes measured in those assigned to these trajectories (Table 3). Interestingly, despite less pack-years smoke exposure compared to trajectory 6, trajectory 7 has more clinical (MMRC, 6MWD, BODE) and radiologic (Perc15, Pi10) impairment, suggesting these individuals are comparatively more susceptible to smoke exposure (Table 3). Despite these notable differences, we did not observe a statistically significant difference in a/l ratios between these two trajectories. Nevertheless, it is interesting to put observations of these trajectories in the context of Smith et al. who observed that participants with established COPD and smaller a/l ratio values had comparable lung function decline as community-based samples, while those with established COPD and larger a/l ratio values had faster lung function decline.^6^ They suggest that the former might correspond to those with low peak lung function in early adulthood followed by normal decline while the latter might correspond to those with persistent accelerated decline, two distinct patterns leading to COPD as described by Lange et al.^2^ Given the similarity in CT characteristics and the pattern of rapid FVC decline shared by trajectories 6 and 7, we raise the possibility that those with the most pronounced dysanapsis correspond to rapid lung function decline patterns. In the case of trajectory 6, rapid decline is evident during COPDGene's period of observation. In the case of trajectory 7, which has patterns of FEV1 decline comparable to the reference trajectory, the period of rapid decline may have occurred earlier in life and may have also been accompanied with failure to achieve peak lung function. On the other hand, the airway predominant trajectories (3, 4, and 5) all have patterns of FEV1 and FVC change comparable the reference trajectory, albeit with more consistently lower Z-score values. These trajectories have less pronounced dysanapsis, and we posit that they correspond to low peak-lung function with more normal rates of decline throughout adulthood. Interestingly, the airway-predominant trajectories have a proportionally greater representation of African Americans compared to the supranormal, reference, and mixed airway and emphysema trajectories.

Smith et al. identified CT-assessed dysanapsis as a risk factor for COPD.^6^ One possible explanation for the trend in a/l ratio values across trajectories observed in Fig. 2 is the differences in COPD prevalence. However, we observe a similar relationship between a/l ratio values and trajectories when we consider only those participants with COPD in each trajectory (Figure E6). Our work shows that dysanapsis is not only a risk-factor for COPD, but the degree of dysanapsis appears to portend distinct trajectories leading to COPD. This is particularly notable for the airway predominant trajectories, which exhibit higher FEV1/FVC ratios in the pre-COPD state. CT-assessed dysanapsis has been shown to be associated with decreased FEV1/FVC in healthy never-smokers during early adulthood.^27^ Our work suggests that individuals with airway under-sizing dysanapsis—independent of decreased FEV1/FVC—may nonetheless be at increased risk for COPD via airway predominant trajectories.

Our work has important clinical implications. First, we demonstrate in the baseline-only cohort that our trajectory model can assign individuals to trajectories using single time point measures of spirometry and assessment of smoking history and status; with additional longitudinal measurements, the accuracy of this assignment is expected to improve. These assignments can be used for risk assessment and management decisions (e.g. by differentiating between airway predominant and mixed airway and emphysema patterns, and by assigning risk for adverse outcomes). Second, our study extends previous research by highlighting the significance of subtle airway under-sizing dysanapsis in relation to airway predominant trajectories. A venue for applying these findings is within CT lung cancer screening cohorts to identify individuals at risk for COPD via distinct trajectories. Strategies that combine CT-assessed dysanapsis and spirometry could further improve early trajectory assignment and enable intervention prior to costly and burdensome outcomes. Furthermore, by assigning individuals to distinct at-risk trajectories, clinical trials targeting specific pathological mechanisms could be enriched.

A strength of our study is the use of the COPDGene study, a large, longitudinal, well-characterized cohort of smokers that is enriched for COPD. This enabled us to identify several distinct lung-function progression patterns leading to COPD with a granularity difficult to achieve with population-based cohorts. Indeed, even with enrichment for COPD, trajectories 5, 6, and 7, account for 4.9%, 2.5%, and 2.7% of the data sample, respectively.

Our study has certain limitations. First, we assume that dysanapsis is a static feature of an individual's lung architecture; however, lack of CT measures at earlier ages is a limitation, and further investigation in younger populations is needed. Nevertheless, our cross-sectional analysis provides no compelling evidence that the CT measure of dysanapsis is associated with age (see supplement, Table E7 and Figures E7–E12). Second, we assessed dysanapsis in a population of smokers with and without COPD. It is reasonable to suspect that the a/l ratio measure could be susceptible to certain disease processes: airway lumen narrowing due to wall thickening and lung hyperinflation due to emphysema. However, our cross-sectional analysis considered both these measures, and we found no compelling evidence to support the notion that these processes affect our findings (see supplement, Table E7). This is in line with the analysis by Smith et al. who concluded that airway to lung ratio measures were unlikely to be affected by emphysema-associated loss of airway tethering, airway remodeling, or lung hyperinflation given that their findings were similar after adjusting for emphysema severity.^6^ Third, COPDGene enrollment criteria required participants to be at least 45 years old at baseline. Hence, we did not have data in early adulthood that could more definitively link trajectories to low-peak lung function and/or early rapid decline that has been observed previously. We mitigated the effect of this data limitation by incorporating an assumption about the peak lung function distribution. Nevertheless, further study of the connection between dysanapsis and lung function trajectories in early life is warranted. This holds particularly true for trajectories 6 and 7, where nearly all members in COPDGene were diagnosed with COPD at baseline. Improving our understanding of the pre-COPD characteristics of these trajectories would significantly improve the ability to assign individuals to them early and thus their clinical utility. Fourth, the baseline-only cohort has fundamental differences compared to the cohort of participants on which the trajectory model was trained (e.g., in terms of overall mortality and exacerbation rates). As such, it is an imperfect cohort on which to validate our findings. Nonetheless, we observe similar patterns in this group of participants, including exacerbation incident ratios, mortality hazard ratios, a/l ratio trends, and CT characteristics (Perc15 and Pi10) with respect to the reference trajectory. Fifth, our analysis was performed in a single cohort; although COPDGene is large and well-characterized, replication in other cohorts is needed to confirm the trajectories we identified as well as their associations to all-cause mortality, exacerbations, and a/l ratios. Last, we used race-adjusted equations to compute FEV1 and FVC Z-scores. However, race-adjusted equations are currently receiving intense scrutiny as their shortcomings are coming to light. Very recent recommendations call for race-neutral or multiethnic equations to be used until more research can be conducted.^28^^,^^29^ Interestingly, Regan et al. found that using non-Hispanic white reference equations tended to reclassify African Americans in the COPDGene study from the GOLD 0 category into the PRISm category (those with preserved FEV1/FVC but with impaired spirometry as measured by FEV1 percent predicted).^28^ We previously noted a greater proportion of African Americans in the airway-predominant trajectories (3–5); given Regan et al.‘s analysis, these proportions may be an underestimate. The factors contributing to proportionally greater representation of African Americans in airway-predominant vs. mixed airway and emphysema trajectories is a fascinating area for further study.

Our findings help close the knowledge gap between the phenomenon of dysanapsis and spirometric trajectories related to COPD, showing that dysanapsis does not uniformly differ across at-risk trajectories. Instead, we show the degree of dysanapsis appears to portend different spirometric patterns of progression with distinct structural patterns in COPD. Whether or not dysanapsis is a causal factor for trajectory assignment requires further analysis. However, regardless of causality, we suggest that strategies that include CT-assessed dysanapsis together with spirometric measures of lung function and smoke exposure assessment are likely to further improve trajectory assignment accuracy, thereby improving early detection of those most at risk for adverse outcomes.

## Contributors

Authors J.R., and A.D. designed the study and outlined the contents of the manuscript. J.R. was responsible for the practical conduct of the study, including planning, data coordination, data modeling, data analysis, and manuscript preparation under the supervision of A.D. A.D. accessed and verified the data and performed data analysis. J.P.C. contributed data measurements required for dysanapsis computations. All authors contributed to data interpretation and manuscript revision prior to its submission, and all authors had the final responsibility to submit for publication.

## Data sharing statement

Immediately after publication, per-subject trajectory assignments will be provided to the COPDGene data coordinating center, which should be the point of contact for researchers interested in this and other COPDGene data. The trajectory model described in our study as well as detailed provenance information will be provided without restriction; requests should be submitted to the corresponding author.

## Declaration of interests

Dr. Ross reports grants from National Heart Lung and Blood Institute, during the conduct of the study. Dr. San José Estepar reports grants from NHLBI, during the conduct of the study; other from Lung Biotechnology, from Insmed, grants from Boehringer Ingelheim, outside the submitted work; and co-founder and stock holder of Quantitative Imaging Solutions, an imaging analytics company in the lung cancer space. Dr. Ash reports grants from NHLBI, during the conduct of the study; other from Quantitative Imaging Solutions, other from Verona Pharmaceuticals, other from Vertex Pharmaceuticals, other from Triangulate Knowledge, other from Boehringer Ingelheim, outside the submitted work. Dr. Pistenmaa reports grants from NIH/NHLBI, during the conduct of the study. Dr. Han reports grants from NIH NHLBI, during the conduct of the study; grants from NIH, personal fees from Sanofi, personal fees from Novartis, personal fees from Nuvaira, personal fees from Sunovion, personal fees from Gala Therapeutics, grants from COPD Foundation, personal fees from AstraZeneca, grants from American Lung Association, personal fees from Boehringer Ingelheim, personal fees from Biodesix, personal fees from GlaxoSmithKline, personal fees from Pulmonx, personal fees from Teva, personal fees from Verona, personal fees from Merck, personal fees from Mylan, personal fees from DevPro, personal fees from Aerogen, personal fees from Polarian, personal fees from United Therapeutics, personal fees from Regeneron, personal fees from Altesa BioPharma, personal fees from Amgen, personal fees from Roche, personal fees from Cipla, personal fees from Chiesi, personal fees from Medscape, personal fees from Integrity, personal fees from NACE, personal fees from Medwiz, outside the submitted work. Novartis, Medtronic (participation on data safety monitoring board/advisory board)—funds paid to institution. Leadership/fiduciary role on the following: COPD Foundation Board, COPD Foundation Scientific Advisory Committee, ALA advisory committee, American Thoracic Society journal editor, ALA volunteer spokesperson, GOLD scientific committee, Emerson School Board (Ann Arbor, MI). Stock or stock options: Meissa Vaccines, Altesa BioPharma. Writing support: GSK, Boehringer Ingelheim, AstraZeneca, Novartis. Royalties from Uptodate, Norton Publishing, and Penguin Random House. Dr. Bhatt reports grants and personal fees from Sanofi, grants and personal fees from Regeneron, personal fees from Boehringer Ingelheim, personal fees from GSK, outside the submitted work. Dr. Bodduluri has nothing to disclose. Dr. Sparrow has nothing to disclose. Dr. Charbonnier reports personal fees and other from Thirona, outside the submitted work. Dr. Washko reports grants from NHLBI, grants from Boehringer Ingelheim, grants from DoD, other from Vertex Pharmaceuticals, other from Pieris Therapeutics, other from Intellia Therapeutics, other from Sanofi, outside the submitted work; and Dr. Washko is a co-founder and equity share holder in Quantitative Imaging Solutions, a company that provides consulting services for image and data analytics. Dr. Washko's spouse works for Biogen. Dr. Diaz reports grants from National Heart Lung and Blood Institute, during the conduct of the study; personal fees from Boehringer Ingelheim, outside the submitted work; in addition, Dr. Diaz has a patent “Methods and Compositions Relating to Airway Dysfunction” pending.

## Appendix A Supplementary data

Supplement clean

Posterior estimates and CIs
