# Supplementary material for: Dysanapsis is differentially related to lung function trajectories with distinct structural and functional patterns in COPD and variable risk for adverse outcomes
Source: eClinicalMedicine. 2024 Jan 5;68:102408. doi: 10.1016/j.eclinm.2023.102408 (PMC10809101; doi:10.1016/j.eclinm.2023.102408)
Supplement: Supplement clean [file mmc1.docx]

**Dysanapsis is Differentially Related to Lung Function Trajectories with Distinct Structural and Functional Patterns in COPD and Variable Risk for Adverse Outcomes: Supplement**

James C. Ross, Raul San Jose Estépar, Sam Ash, Carrie Pistenmaa, MeiLan Han, Surya P. Bhatt, Sandeep Bodduluri, David Sparrow, Jean-Paul Charbonnier, George R. Washko, Alejandro A. Diaz

**Data Selection**

**Figure E1**. Data selection procedure.

**Trajectory Analysis**

Our Bayesian trajectory approach used a combination of prior knowledge, data-driven inference, and model selection based on clinical relevance (using analysis of mortality and exacerbation risk). We took advantage of prior work that establishes z-score values for FEV1 and FVC as functions of age, height, sex, and race (1). By centering the age variable in our trajectory analysis (at 20 for men and 18 for women), the trajectory intercept term is then naturally interpreted as the (FEV1 and FVC) value achieved in early adulthood. The Bayesian paradigm allows us to incorporate prior belief in the trajectory modeling using *priors*. Commonly used likelihood-based trajectory approaches do not permit incorporation of prior knowledge into the data analysis (2,3). By using a standard normal distribution for the intercept prior, we exploited prior knowledge of peak FEV1 and FVC Z-score values, and we also mitigated the effects of data absence in early adulthood (due to COPDGene enrollment criteria requiring participants to be at least 45 years old). We chose the variances of the priors over the intercept terms to restrict the intercept posteriors to be within -3 and 3 (i.e. three standard deviations of the expected mean value of zero) to enforce our prior belief about the range of reasonably possible intercept values. To do this, we performed exploratory analysis by iteratively running the trajectory algorithm and gradually reducing the intercept prior variances until we observed that intercept posteriors were within the expected range.

We also used zero-centered normal distributions as priors for the other predictors in the trajectory model to reflect a null hypothesis of a predictor having no effect. We used a visual feedback routine provided in the *bayes_traj* software distribution to visualize trajectories corresponding to random draws from the coefficient priors super-imposed on the COPDGene data. We iteratively adjusted coefficient prior variances and visualized random trajectory draws until we observed that random draws corresponded to vague but plausible priors (i.e. random trajectory draws tending to lie within the observed data sample range without too many highly implausible trajectories).

The number of trajectories is determined by both the data and the Bayesian priors selected for the model variables. The priors that have the greatest effect on the number of trajectories are those for the FEV1 and FVC Z-score residual variances: if the practitioner indicates with these priors that the residual variance is expected to be small, the algorithm will tend to identify a greater number of trajectories (and vice versa). Our model selection strategy included considering a range of plausible priors over the variances.

We also note that our Bayesian trajectory algorithm is *nonparametric*. Bayesian nonparametric algorithms are a category of approaches that adjust in complexity to best explain the data. Here this means that – influenced by the priors – the data drives the number of detected trajectories. We focus on those trajectories that account for at least 2% of the data sample. Other, smaller trajectories can also be detected; these arise when the algorithm is unable to assign an individual or small group of individuals to one of the other trajectories; in those cases, the algorithm spawns a new trajectory to explain those observations.

We considered several alternative predictor sets, and for each predictor set, we performed Bayesian trajectory analysis across a range of plausible prior settings for the FEV1 and FVC residual variances. The predictor sets we considered were:

- intercept, years since presumed peak lung function, (years since presumed peak lung function)^2^, pack-years smoke exposure, (pack-years smoke exposure) ^2^, (current smoking status)X(years since presumed peak lung function)
- intercept, years since presumed peak lung function, (years since presumed peak lung function)^2^, pack-years smoke exposure, (current smoking status)X(years since presumed peak lung function)
- intercept, years since presumed peak lung function, pack-years smoke exposure, (current smoking status)X(years since presumed peak lung function)
- intercept, years since presumed peak lung function, pack-years smoke exposure

The *bayes_traj* (<https://github.com/acil-bwh/bayes_traj>) software routine we deployed in our experiments uses a form of approximate Bayesian estimation known as variational inference, which treats inference as an optimization problem. As is common with optimizers, they must be initialized. We used 300 random restarts with 500 optimizer iterations per restart for each predictor set and prior setting. We used a version of the Watanable-Akaike information criterion (WAIC2) to evaluate model fits; lower WAIC2 scores indicate better fits (4). Table E1 shows the WAIC2 scores corresponding to the best fit for each predictor set and prior.

**Table E1. WAIC2 scores of best trajectory model fits corresponding to predictor sets and residual variance priors considered in our experiments.**


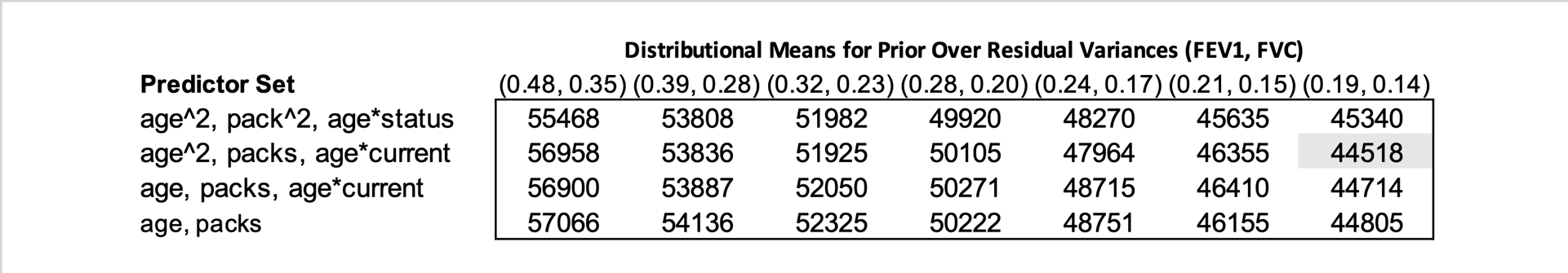


Columns indicate mean values for FEV1 and FVC Z-score residual variance priors. The cell shaded gray indicates the best WAIC2 score among the predictor sets and prior settings we considered. All predictor sets included an intercept term. age=years since presumed peak lung function, packs=pack years smoke exposure, current=current smoking status. The first two models additionally include age (years since presumed peak lung function) as a predictor.

As Table E1 indicates, the best data fit corresponded to the predictor set years since presumed peak lung function, (years since presumed peak lung function)^2^, packs, (years since presumed peak lung function)*current, and an intercept term with priors over FEV1 and FVC residual variances having means of 0.19 and 0.14, respectively. Next, we inspected all the models corresponding to the 300 random restarts for these predictors and priors. Figure E2 shows WAIC2 scores for each random restart sorted from best (left) to worst (right).


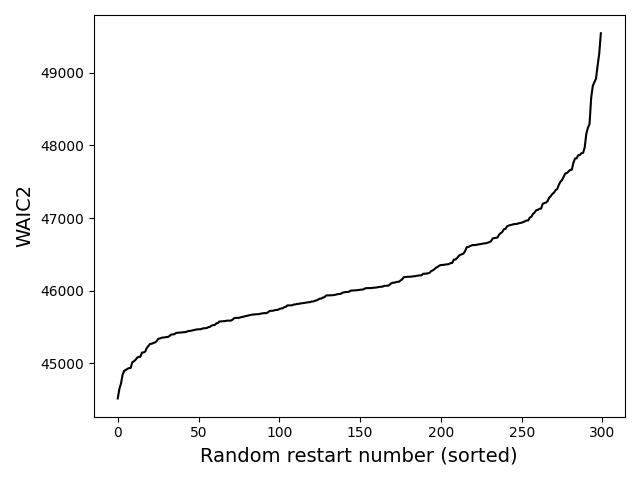


**Figure E2.** WAIC2 scores corresponding to the 300 random restarts using predictors: intercept, (age centered)^2^, pack years smoke exposure, and (age centered)*(current smoking status) and priors over residual variances with means 0.19 (FEV1) and 0.14 (FVC). The random restart ordering has been sorted from smallest to largest.

The top results are qualitatively similar and align with our prior expectation of trajectories varying by both rates of decline and projected peak lung function obtained in early adulthood. We restricted our analysis to those trajectories accounting for at least 2% of the data sample; with this criterion, the best fit model describes eight trajectories; the fifth best fit model describes seven. We compared these two models using exacerbation and mortality analysis, in each case iteratively changing the reference trajectory to assess the meaningfulness of each trajectory partitioning. We observed statistically significant discrimination between adjacent trajectories in terms of mortality hazard ratios in the model with seven trajectories but not in the model with eight. (The two models do not differ with respect to their exacerbation incident risk ratio discrimination). We therefore chose the model with seven trajectories as the focus of the main manuscript. Figure E3 shows FEV1 and FVC Z-scores vs years since presumed peak lung function. Tables E2 – E5 show HRs and IRRs for the final model, and Figure E4 shows a Kaplan-Meier plot of survival probabilities vs age.


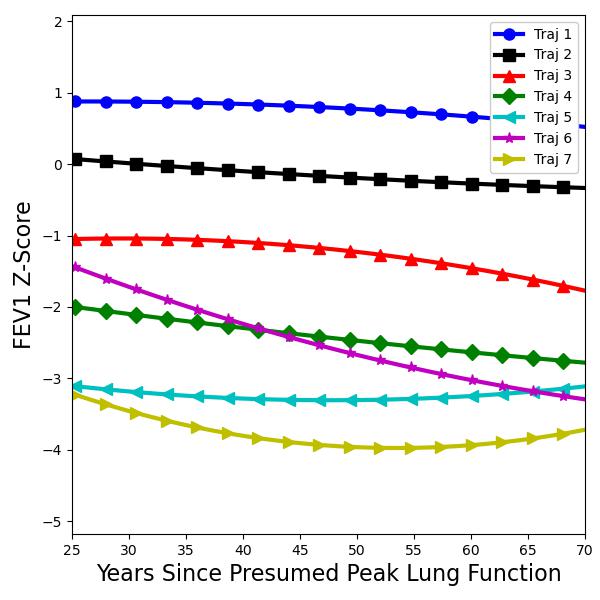

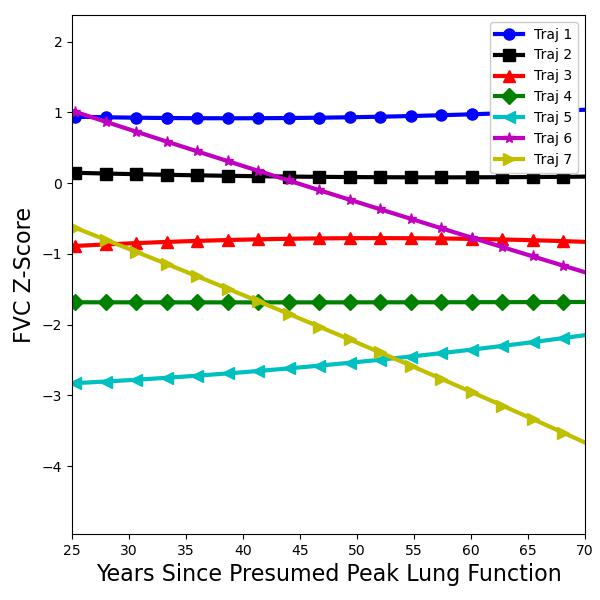


**Figure E3.** FEV1 (left) and FVC (right) Z-Score values as a function of years since presumed peak lung function for each of the trajectories analyzed in our study.

**Table E2. Hazard ratios for all-cause mortality and incident rate ratios for the total number of exacerbations during follow-up by trajectory with trajectory 3 treated as the reference trajectory.**

|  | Reduced Models | | | | Full Models | | | |
| --- | --- | --- | --- | --- | --- | --- | --- | --- |
| **Traj** | **HR** | **p-value** | **IRR** | **p-value** | **HR** | **p-value** | **IRR** | **p-value** |
| 1 | 0.60 | 0.03 | 0.53 | < 0.001 | 0.79 | 0.31 | 0.60 | < 0.001 |
| 2 | 0.83 | 0.16 | 0.61 | < 0.001 | 0.94 | 0.65 | 0.67 | < 0.001 |
| 4 | 2.08 | < 0.001 | 2.05 | < 0.001 | 1.39 | 0.005 | 1.75 | < 0.001 |
| 5 | 3.60 | < 0.001 | 3.93 | < 0.001 | 1.86 | < 0.001 | 2.85 | < 0.001 |
| 6 | 2.46 | < 0.001 | 3.01 | < 0.001 | 1.63 | 0.02 | 2.41 | < 0.001 |
| 7 | 6.18 | < 0.001 | 6.73 | < 0.001 | 2.06 | < 0.001 | 4.44 | < 0.001 |

Left: extended Cox models adjusted for pack-years smoke exposure, current smoking status, sex, and race; zero-inflated negative binomial mixed models adjusted for age, pack-years smoke exposure, current smoking status, sex, and race. Right: extended Cox models adjusted for pack-years smoke exposure, current smoking status, BMI, MMRC, 6MWD, sex, and race; zero-inflated negative binomial mixed models adjusted for age, pack-years smoke exposure, current smoking status, SGRQ, GERD, number of exacerbations in the previous year, sex, and race.

**Table E3. Hazard ratios for all-cause mortality and incident rate ratios for the total number of exacerbations during follow-up by trajectory group membership with trajectory 4 treated as the reference trajectory.**

| **Traj** | **HR** | **p-value** | **IRR** | **p-value** | **HR** | **p-value** | **IRR** | **p-value** |
| --- | --- | --- | --- | --- | --- | --- | --- | --- |
| 1 | 0.29 | < 0.001 | 0.26 | < 0.001 | 0.56 | 0.02 | 0.34 | < 0.001 |
| 2 | 0.40 | < 0.001 | 0.30 | < 0.001 | 0.67 | 0.004 | 0.38 | < 0.001 |
| 3 | 0.48 | < 0.001 | 0.49 | < 0.001 | 0.71 | 0.005 | 0.57 | < 0.001 |
| 5 | 1.73 | < 0.001 | 1.92 | < 0.001 | 1.34 | 0.08 | 1.63 | < 0.001 |
| 6 | 1.18 | 0.41 | 1.47 | 0.02 | 1.17 | 0.45 | 1.38 | 0.03 |
| 7 | 2.97 | < 0.001 | 3.29 | < 0.001 | 1.48 | 0.03 | 2.54 | < 0.001 |

Left: extended Cox models adjusted for pack-years smoke exposure, current smoking status, sex, and race; zero-inflated negative binomial mixed models adjusted for age, pack-years smoke exposure, current smoking status, sex, and race. Right: extended Cox models adjusted for pack-years smoke exposure, current smoking status, BMI, MMRC, 6MWD, sex, and race; zero-inflated negative binomial mixed models adjusted for age, pack-years smoke exposure, current smoking status, SGRQ, GERD, number of exacerbations in the previous year, sex, and race.

**Table E4. Hazard ratios for all-cause mortality and incident rate ratios for the total number of exacerbations during follow-up by trajectory group membership with trajectory 5 treated as the reference trajectory.**

| **Traj** | **HR** | **p-value** | **IRR** | **p-value** | **HR** | **p-value** | **IRR** | **p-value** |
| --- | --- | --- | --- | --- | --- | --- | --- | --- |
| 1 | 0.17 | < 0.001 | 0.13 | < 0.001 | 0.42 | 0.002 | 0.21 | < 0.001 |
| 2 | 0.23 | < 0.001 | 0.16 | < 0.001 | 0.51 | < 0.001 | 0.24 | < 0.001 |
| 3 | 0.28 | < 0.001 | 0.25 | < 0.001 | 0.54 | < 0.001 | 0.35 | < 0.001 |
| 4 | 0.58 | < 0.001 | 0.52 | < 0.001 | 0.75 | 0.08 | 0.61 | < 0.001 |
| 6 | 0.68 | 0.10 | 0.77 | 0.14 | 0.88 | 0.59 | 0.84 | 0.32 |
| 7 | 1.72 | 0.005 | 1.71 | 0.002 | 1.10 | 0.64 | 1.56 | 0.006 |

Left: extended Cox models adjusted for pack-years smoke exposure, current smoking status, sex, and race; zero-inflated negative binomial mixed models adjusted for age, pack-years smoke exposure, current smoking status, sex, and race. Right: extended Cox models adjusted for pack-years smoke exposure, current smoking status, BMI, MMRC, 6MWD, sex, and race; zero-inflated negative binomial mixed models adjusted for age, pack-years smoke exposure, current smoking status, SGRQ, GERD, number of exacerbations in the previous year, sex, and race.

**Table E5. Hazard ratios for all-cause mortality and incident rate ratios for the total number of exacerbations during follow-up by trajectory group membership with trajectory 6 treated as the reference trajectory.**

| **Traj** | **HR** | **p-value** | **IRR** | **p-value** | **HR** | **p-value** | **IRR** | **p-value** |
| --- | --- | --- | --- | --- | --- | --- | --- | --- |
| 1 | 0.24 | < 0.001 | 0.18 | < 0.001 | 0.48 | 0.01 | 0.25 | < 0.001 |
| 2 | 0.34 | < 0.001 | 0.20 | < 0.001 | 0.58 | 0.01 | 0.28 | < 0.001 |
| 3 | 0.41 | < 0.001 | 0.33 | < 0.001 | 0.61 | 0.02 | 0.42 | < 0.001 |
| 4 | 0.84 | 0.41 | 0.68 | 0.02 | 0.85 | 0.5 | 0.73 | 0.03 |
| 5 | 1.46 | 0.10 | 1.31 | 0.14 | 1.14 | 0.6 | 1.18 | 0.3 |
| 7 | 2.51 | < 0.001 | 2.24 | < 0.001 | 1.26 | 0.4 | 1.85 | 0.001 |

Left: extended Cox models adjusted for pack-years smoke exposure, current smoking status, sex, and race; zero-inflated negative binomial mixed models adjusted for age, pack-years smoke exposure, current smoking status, sex, and race. Right: extended Cox models adjusted for pack-years smoke exposure, current smoking status, BMI, MMRC, 6MWD, sex, and race; zero-inflated negative binomial mixed models adjusted for age, pack-years smoke exposure, current smoking status, SGRQ, GERD, number of exacerbations in the previous year, sex, and race.


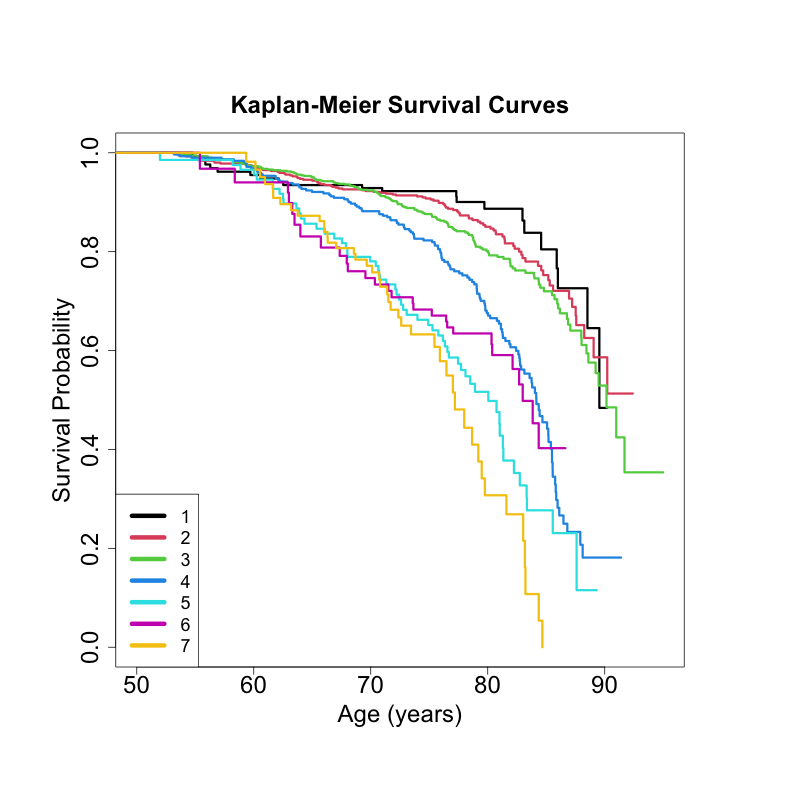


**Figure E4.** Kaplan-Meier plot of survival probabilities as a function of age for the seven trajectories analyzed in our study.

**Table E6. Characteristics of COPDGene participants by lung function trajectory in the baseline-only cohort.**

| **Characteristic** | **1** | **2** | **3** | 4 | **5** | **6** | **7** |
| --- | --- | --- | --- | --- | --- | --- | --- |
| *N* | 217 (5.8) | 791 (21.0) | 994 (26.4) | 689 (18.3) | 199 (5.3) | 119 (3.2) | 185 (4.9) |
| Female | 64 (29) | 321 (40) | 393 (39) | 303 (43) | 75 (37) | 51 (42) | 78 (42) |
| African American | 109 (50) | 369 (46) | 442 (44) | 304 (44) | 79 (39) | 18 (15) | 39 (21) |
| Age, yr | 56 ± 8 | 57 ± 9 | 57 ± 9 | 59 ± 10 | 61 ± 9 | 66 ± 10 | 62 ± 8 |
| FEV1 Z-Score | 0.9 ± 0.5 | -0.1 ± 0.5 | -1.2 ± 0.6 | -2.3 ± 0.7 | -3.4 ± 0.6 | -2.8 ± 0.6 | -3.9 ± 0.5 |
| FEV1 % | 113 ± 7 | 98 ± 9 | 82 ± 11 | 62 ± 14 | 43 ± 13 | 52 ± 14 | 34 ± 11 |
| FVC Z-Score | 1.0 ± 0.4 | 0.1 ± 0.4 | -0.8 ± 0.4 | -1.7 ± 0.4 | -2.8 ± 0.4 | -0.2 ± 0.5 | -1.6 ± 0.6 |
| FVC % | 114 ± 7 | 100 ± 7 | 87 ± 7 | 73 ± 7 | 57 ± 7 | 94 ± 9 | 73 ± 11 |
| FEV1/FVC Z-Score | -0.2 ± 0.9 | -0.4 ± 1.0 | -0.7 ± 1.2 | -1.5 ± 1.6 | -2.3 ± 1.7 | -3.8 ± 0.7 | -4.4 ± 0.6 |
| BMI, kg/m2 | 27 ± 5 | 28 ± 6 | 29 ± 6 | 30 ± 7 | 31 ± 8 | 25 ± 5 | 26 ± 6 |
| Pack-years | 41 ± 22 | 43 ± 25 | 43 ± 25 | 46 ± 27 | 52 ± 27 | 47 ± 22 | 49 ± 22 |
| Pi10 | 2.0 ± 0.5 | 2.1 ± 0.5 | 2.3 ± 0.5 | 2.7 ± 0.6 | 3.0 ± 0.6 | 2.7 ± 0.5 | 3.0 ± 0.5 |
| Perc15 | -911 ± 20 | -906 ± 24 | -902 ± 28 | -906 ± 36 | -915 ± 40 | -950 ± 24 | -953 ± 26 |
| 6MWD (feet) | 1525 ± 355 | 1432 ± 338 | 1355 ± 369 | 1168 ± 394 | 970 ± 431 | 1229 ± 349 | 1030 ± 382 |
| BODE | 0.7 ± 1.0 | 0.8 ± 1.2 | 1.2 ± 1.5 | 2.8 ± 2.2 | 4.9 ± 2.3 | 3.4 ± 2.1 | 5.6 ± 2.0 |
| MMRC | 0.8 ± 1.2 | 0.9 ± 1.3 | 1.2 ± 1.4 | 1.9 ± 1.5 | 2.7 ± 1.3 | 2.3 ± 1.3 | 2.8 ± 1.0 |
| CT Lung Vol. | 6.10 ± 1.4 | 5.51 ± 1.2 | 5.14 ± 1.2 | 5.05 ± 1.4 | 5.32 ± 1.4 | 6.69 ± 1.5 | 6.47 ± 1.5 |
| COPD | 21 (9) | 147 (18) | 305 (30) | 348 (50) | 142 (71) | 119 (100) | 185 (100) |

Data are presented as mean ± SD or number (percent). Trajectories 3-5 (shaded light blue) are characterized by airway predominant abnormality leading to COPD; trajectories 6 and 7 (shaded light red) are characterized by mixed airway and parenchymal abnormality.


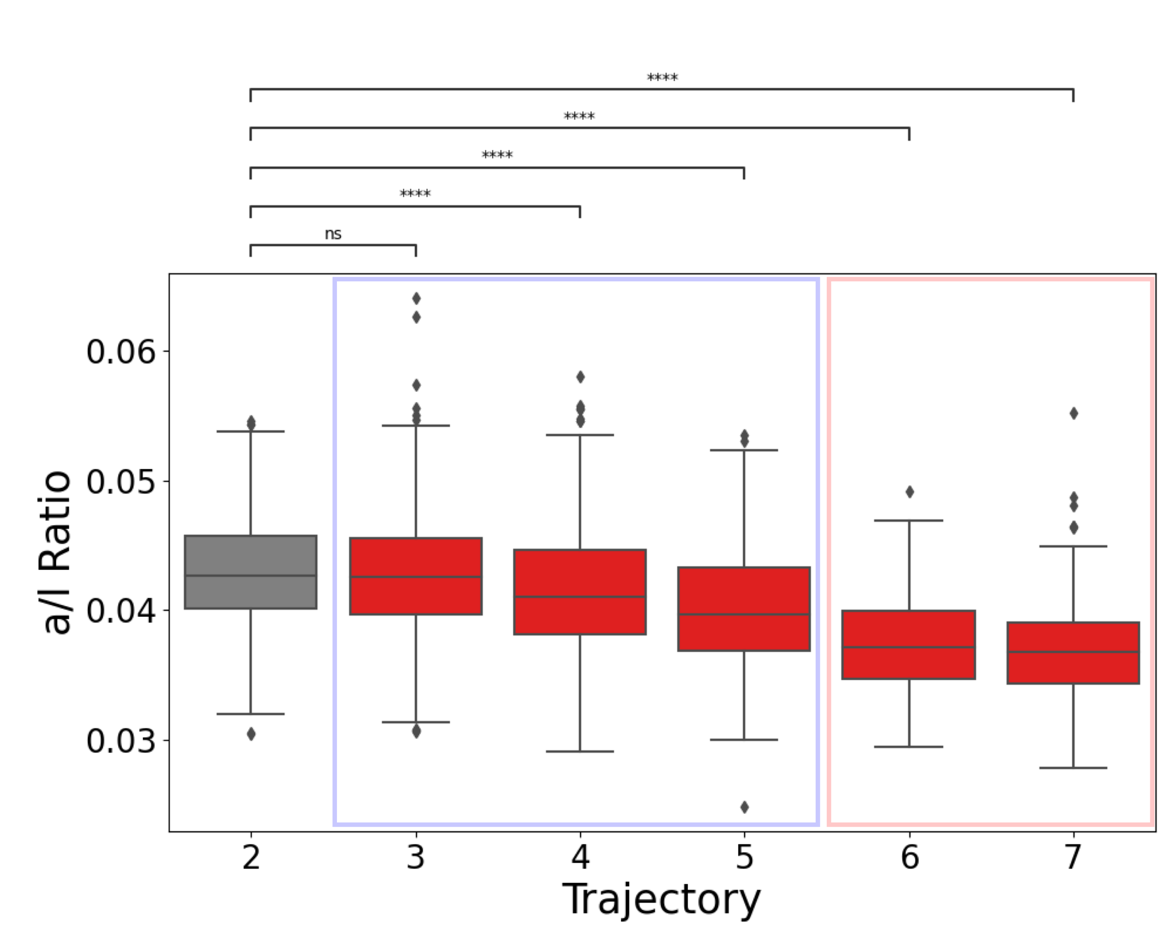


**Figure E5.** Boxplot showing a/l ratios by trajectory for those COPDGene participants in the baseline-only cohort. Reference trajectory 2 in gray and trajectories at increased risk of all-cause mortality and exacerbations (trajectories 3–7) in red. Trajectories 3-5 (light blue border) are characterized by airway predominant abnormality leading to COPD; trajectories 6 and 7 (light red border) are characterized by mixed airway and emphysema abnormality. Indicated above are p-values corresponding to pairwise statistical comparisons between trajectory 2 and each at-risk trajectory (using Mann-Whitney test and Bonferroni correction for multiple comparisons): * p < 0.05, **** p < 0.0001.


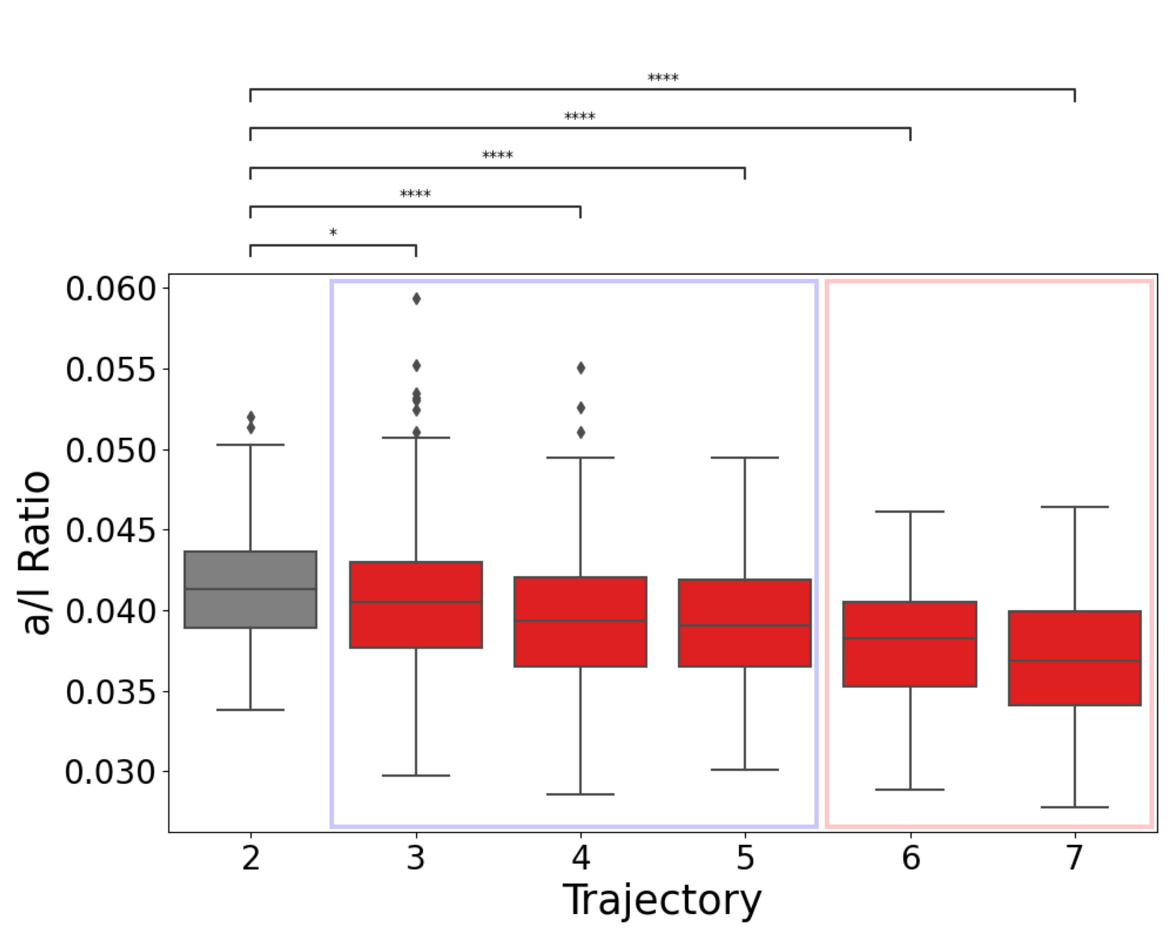


**Figure E6.** Boxplot showing a/l ratios by trajectory, restricted to those COPDGene participants in the modeling cohort with COPD. Reference trajectory 2 in gray and trajectories at increased risk of all-cause mortality and exacerbations (trajectories 3–7) in red. Trajectories 3-5 (light blue border) are characterized by airway predominant abnormality leading to COPD; trajectories 6 and 7 (light red border) are characterized by mixed airway and emphysema abnormality. Indicated above are p-values corresponding to pairwise statistical comparisons between trajectory 2 and each at-risk trajectory (using Mann-Whitney test and Bonferroni correction for multiple comparisons): * p < 0.05, **** p < 0.0001.

**Association analysis of a/l ratio, age, emphysema, and airway wall thickening**

Within each trajectory we performed linear regression between age and a/l ratio, Perc15, and Pi10. We also computed Pearson product-moment correlation coefficients between age and each of the CT biomarkers. Results are summarized in Table E7. To test regression modeling assumptions (linearity, homoscedasticity, and normality of residuals), we plotted regression residuals vs predicted values, residual histograms, and Q-Q plots (Figures E7-E12). Although we note some minor departures from normality in some cases and some subtle evidence of heteroscedasticity, we do not consider these significant violations of ordinary least squares regression assumptions.

For all trajectories, the correlation between a/l ratio and age is negligible. This is true even in trajectories 4-7 which exhibit a low to moderate inverse correlation with Perc15. Thus, this analysis fails to provide compelling evidence that a/l ratio changes with age or that the a/l ratio is significantly affected by airway wall thickening and emphysematous destruction of the parenchyma.­

**Table E7. Analysis of relationship between a/l ratio, Pi10, Perc15 and age**


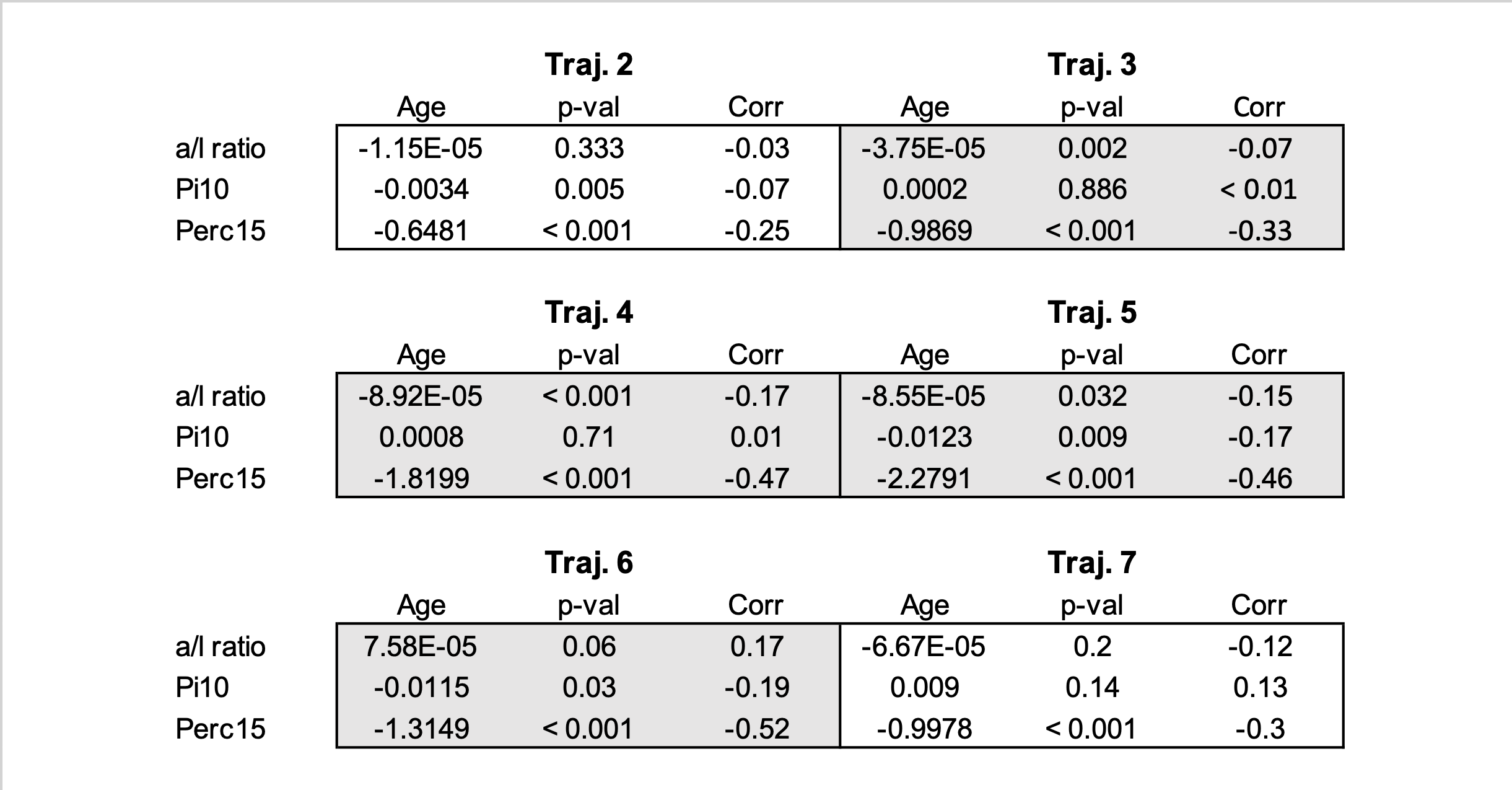


Table provides regression coefficients, p-values, and Pearson product-moment correlation coefficients (Corr) for measures of dysanapsis (a/l ratio), airway wall thickening (Pi10), and emphysema (Perc15). Gray shading highlights those trajectories for which there is a statistically significant relationship between a/l ratio and age.


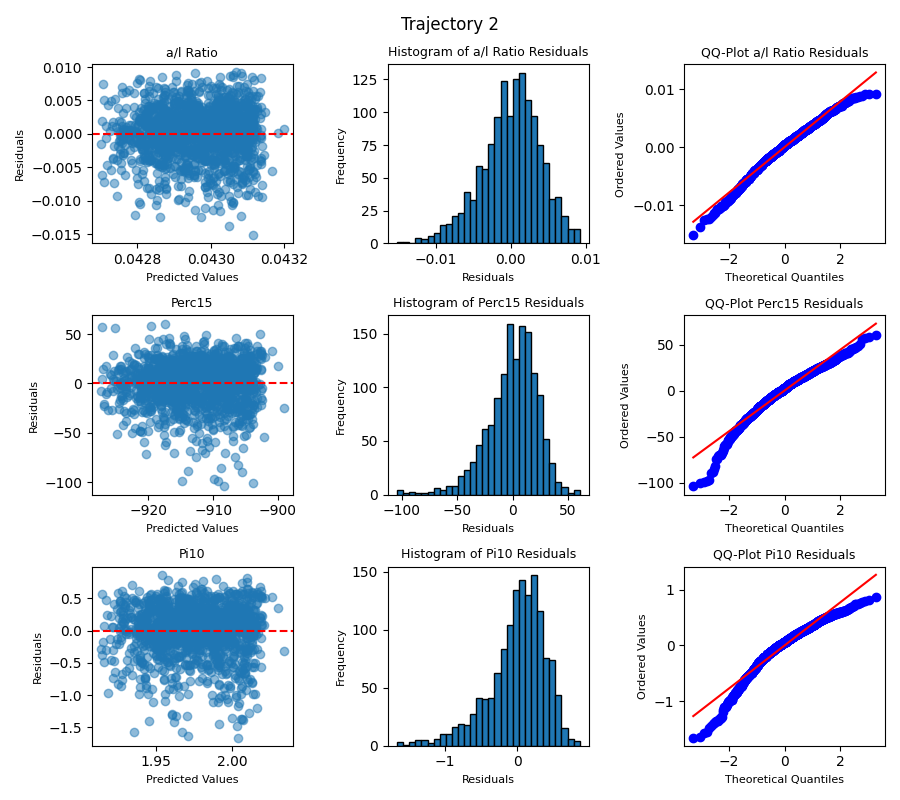


**Figure E7.** Residuals vs predicted values (left column), histogram of residuals (middle column) and Q-Q plot (right column) corresponding to each of the regression models summarized in Table E7 for trajectory 2.


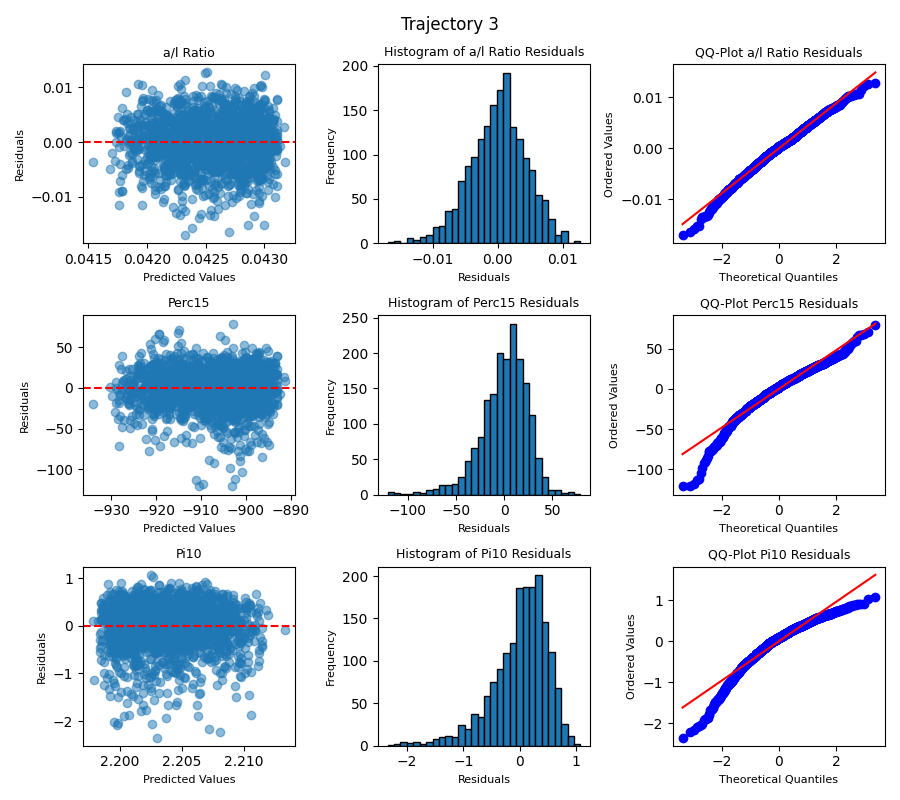


**Figure E8.** Residuals vs predicted values (left column), histogram of residuals (middle column) and Q-Q plot (right column) corresponding to each of the regression models summarized in Table E7 for trajectory 3.


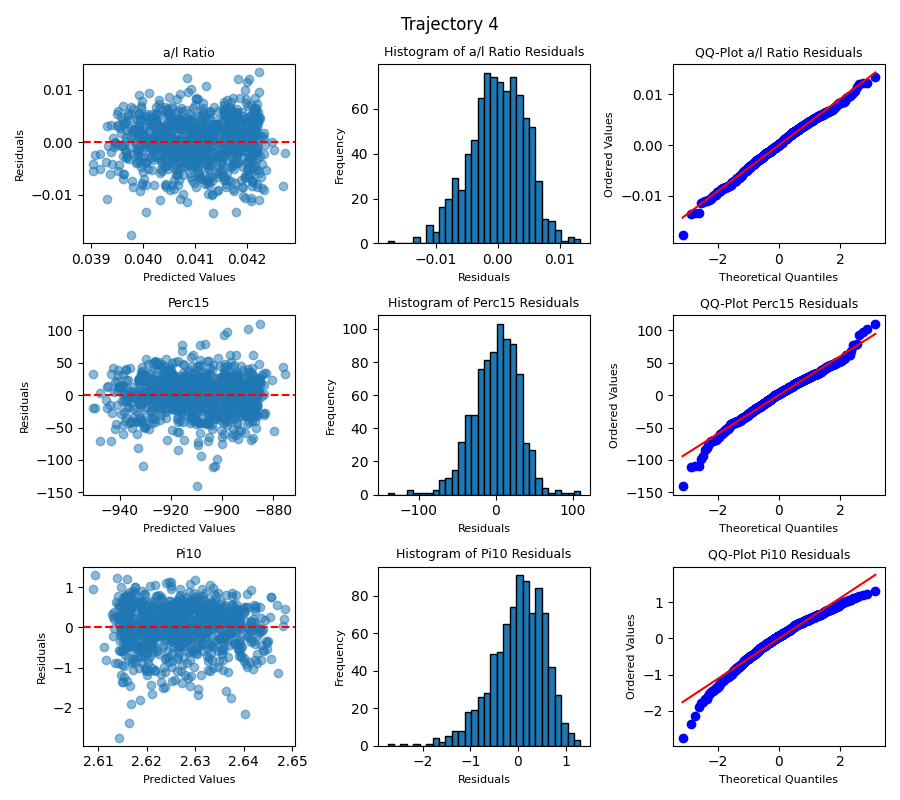


**Figure E9.** Residuals vs predicted values (left column), histogram of residuals (middle column) and Q-Q plot (right column) corresponding to each of the regression models summarized in Table E7 for trajectory 4.


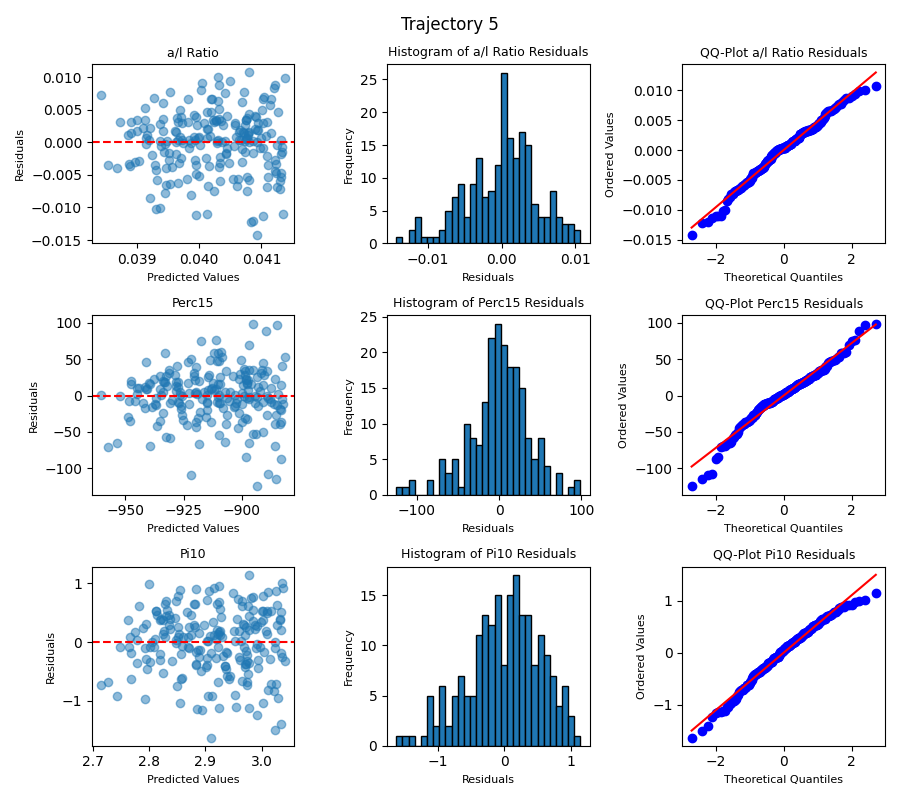


**Figure E10.** Residuals vs predicted values (left column), histogram of residuals (middle column) and Q-Q plot (right column) corresponding to each of the regression models summarized in Table E7 for trajectory 5.


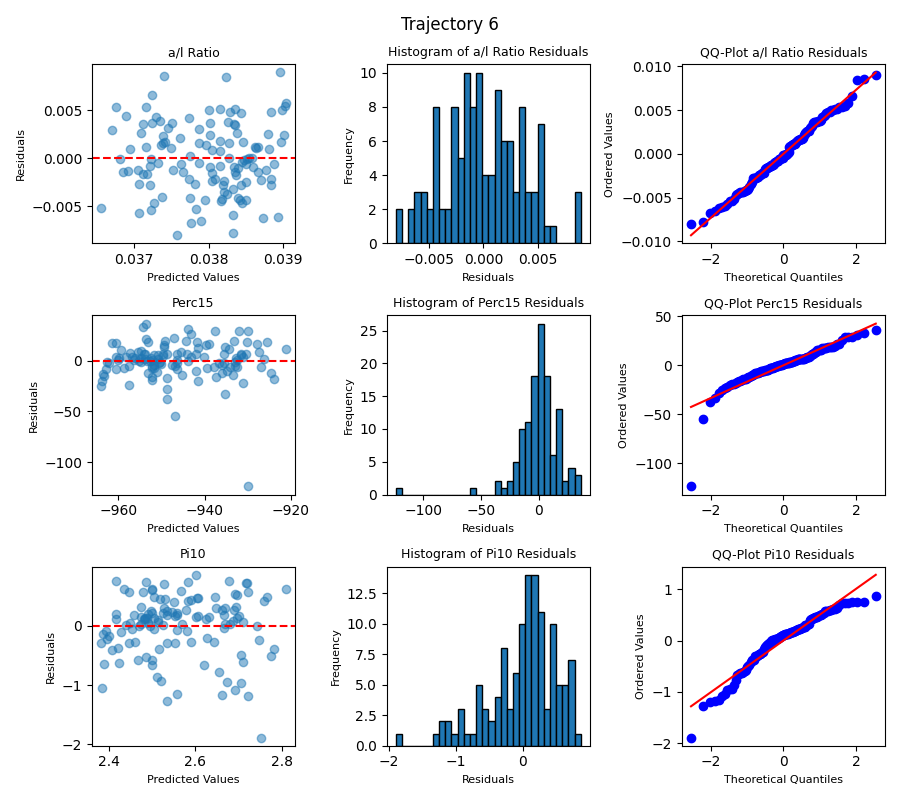


**Figure E11.** Residuals vs predicted values (left column), histogram of residuals (middle column) and Q-Q plot (right column) corresponding to each of the regression models summarized in Table E7 for trajectory 6.


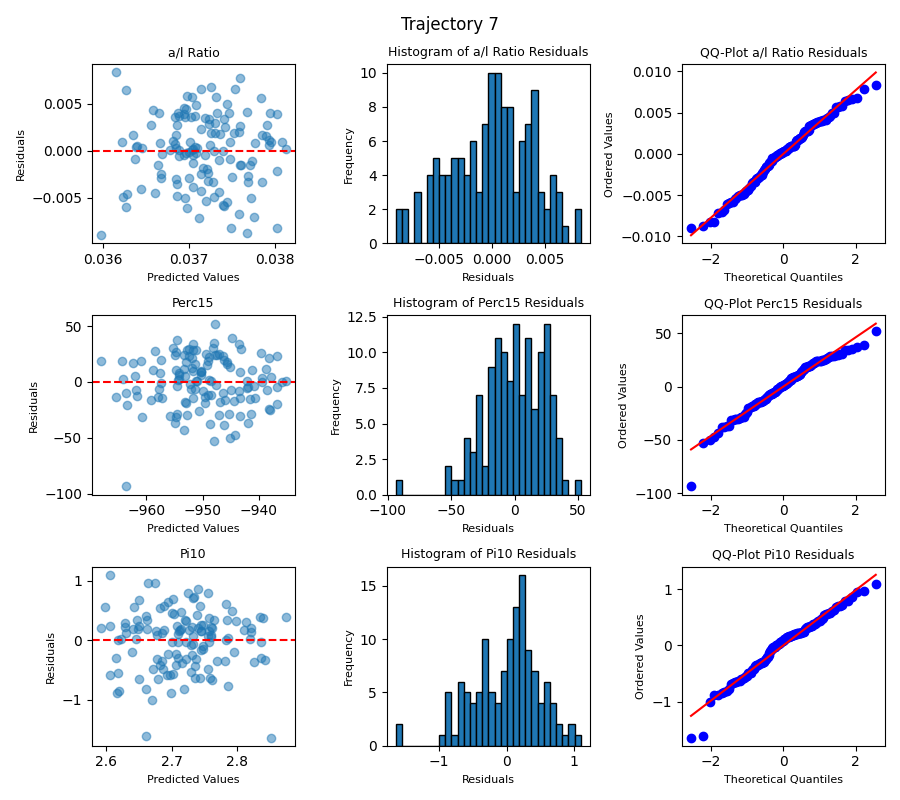


**Figure E12.** Residuals vs predicted values (left column), histogram of residuals (middle column) and Q-Q plot (right column) corresponding to each of the regression models summarized in Table E7 for trajectory 7.

**Bibliography**

E1.Quanjer PH, Stanojevic S, Cole TJ, Baur X, Hall GL, Culver BH, et al. Multi-ethnic reference values for spirometry for the 3-95-yr age range: The global lung function 2012 equations. Eur Respir J. 2012;40(6):1324–43.

E2. Nagin DS. Analyzing Developmental Trajectories : A Semiparametric , Group-Based Approach. Psychological Methods. 4.2 (1999): 139

E3. Proust-lima C, Liquet B. Estimation of Extended Mixed Models Using Latent Classes and Latent Processes : The R Package lcmm.

E4. Watanabe S. Asymptotic Equivalence of Bayes Cross Validation and Widely Applicable Information Criterion in Singular Learning Theory. J Mach Learn Res. 2010 Dec 1;11:3571–94.
